# Causes of stillbirth in sub-Saharan Africa and South Asia: Findings from Child Health and Mortality Prevention Surveillance, 2016–2023

**DOI:** 10.1371/journal.pgph.0005235

**Published:** 2026-10-27

**Authors:** Afruna Rahman, Kyu Han Lee, Shams El Arifeen, Mohammad Zahid Hossain, Atique Iqbal Chowdhury, Muntasir Alam, Afsana Afrin, Ikechukwu Udo Ogbuanu, Solomon Samura, Erick Kaluma, Julius Ojulong, Samba Sow, Adama Mamby Keita, Milagritos D. Tapia, Kiranpreet Kaur Chawla, Karen Kotloff, Shabir A. Madhi, Sana Mahtab, Yasmin Adam, Amy Wise, Ziyaad Dangor, Christopher Mugah, Elizabeth Oele, Richard Omore, Aggrey K. Igunza, Dickens Onyango, Inacio Mandomando, Quique Bassat, Sara Ajanovic, Rosauro Varo, Elisio Xerinda, Nega Assefa, J. Anthony G. Scott, Lola Madrid, Melisachew M. Yeshi, Fikremelekot Temesgen, Lucy Liu, Portia Mutevedzi, Dianna M. Blau, Victor Akelo, Robert F. Breiman, Cynthia G. Whitney, Emily S. Gurley, for the CHAMPS Consortium

**Affiliations:** 1 International Center for Diarrhoeal Diseases Research (icddr, b), Dhaka, Bangladesh; 2 Department of Epidemiology, Johns Hopkins Bloomberg School of Public Health, Baltimore, Maryland, United States of America; 3 Emory Global Health Institute, Emory University, Atlanta, Georgia, United States of America; 4 The Africa Research Collaborative (the ARC), Freetown, Sierra Leone; 5 World Hope International, Makeni, Sierra Leone; 6 Centre pour le Développement des Vaccins (CVD-Mali), Ministère de la Santé, Bamako, Mali; 7 Center for Vaccine Development and Global Health, University of Maryland School of Medicine, Baltimore, Maryland, United States of America; 8 South African Medical Research Council Vaccines and Infectious Diseases Analytics (VIDA) Research Unit, Faculty of Health Sciences, University of the Witwatersrand, Johannesburg, South Africa; 9 Faculty of Health Sciences, Wits Infectious Disease and Oncology Research Institute, University of the Witwatersrand, Johannesburg, South Africa; 10 Wits Department of Obstetrics & Gynaecology, Faculty of Health Sciences, University of the Witwatersrand, Johannesburg, South Africa; 11 Kenya Medical Research Institute (KEMRI), Kisumu, Kenya; 12 Kisumu County of Department of Health, Kisumu, Kenya; 13 Centro de Investigação em Saúde de Manhiça [CISM], Maputo, Mozambique; 14 Instituto Nacional de Saúde [INS], Maputo, Mozambique; 15 ISGlobal - Hospital Clínic, Universitat de Barcelona, Barcelona, Spain; 16 Institutó Catalana de Recerca i Estudis Avançats [ICREA], Barcelona, Spain; 17 Pediatrics Department, Hospital Sant Joan de Déu, Universitat de Barcelona, Esplugues, Barcelona, Spain; 18 CIBER de Epidemiología y Salud Pública, Instituto de Salud Carlos III, Madrid, Spain; 19 College of Health and Medical Sciences, Haramaya University, Harar, Ethiopia; 20 Department of Infectious Disease Epidemiology and International Health, London School of Hygiene & Tropical Medicine, London, United Kingdom; 21 Ayder Specialized Comprehensive Hospital, Mekelle University, Mekele, Ethiopia; 22 Liverpool School of Tropical Medicine (LSTM), Liverpool, England; 23 Addis Ababa University, Addis Ababa, Ethiopia; 24 Center for Global Health, Centers for Disease Control and Prevention, Atlanta, Georgia, United States of America; Yale University, UNITED STATES OF AMERICA

## Abstract

**Background:**

Globally, an estimated 1.9 million stillbirths occur annually, yet significant knowledge gaps exist regarding the causes of stillbirths, particularly in high-burden regions. We investigated fetal and maternal conditions causing stillbirths in seven countries in sub-Saharan Africa and South Asia and described missed opportunities for prevention.

**Methods:**

Child Health and Mortality Prevention Surveillance (CHAMPS) identified stillbirths at sites in Bangladesh, Ethiopia, Kenya, Mali, Mozambique, Sierra Leone, and South Africa. We asked families for consent to conduct minimally invasive tissue sampling (MITS) from December 2016 to December 2023. An expert panel reviewed test results, clinical information, and verbal autopsy data and assigned fetal and maternal conditions leading to stillbirths and identified missed opportunities for prevention.

**Findings:**

A cause of death (fetal or maternal condition) was determined for 94% (2342/2492) of stillbirths; the most frequent condition in the fetus was intrauterine hypoxia (75%, 1864/2492) across all sites, resulting from either maternal condition (49%, 922/1864) or placental causes (33%, 610/1864). Congenital infections were determined to be the cause of 9% (228/2492) of stillbirths, accounting for the highest proportion in South Africa (31%, 96/306). Group B Streptococcus, *Escherichia coli*, and *Enterococcus faecalis* were the most common causative pathogens. Congenital birth defects caused 9% (227/2492) overall and were most common in Ethiopia (24%, 136/568). Primary maternal conditions were identified in 72%, most often placental complications (18%, 446/2492) and maternal hypertension (17%, 414/2492). Complications of placenta, cord and membranes were more common in Mali (43%, 92/212) while maternal medical and surgical conditions were most frequently observed in South Africa (40%, 121/306) and Bangladesh (39%, 158/405). Most (72%, 1808/2492) causes of stillbirth were considered preventable, with heterogeneity observed across sites on the recommended prevention strategies.

**Interpretation:**

Complications of pregnancy or delivery accounted for most stillbirths across all sites, with intrauterine hypoxia identified as the predominant fetal condition highlighting widespread and persistent gaps in the timely identification and management of maternal and placental conditions during antenatal and obstetric care across sites in Africa and South Asia. At the same time, substantial geographic variation in causes and prevention opportunities, including for infections and birth defects, underscored the need for locally tailored strategies. Strengthening facility-based care alongside targeted interventions based on site-specific patterns, will be critical to reducing the stillbirth burden in these high-priority regions.

**Funding:**

The Gates Foundation

## Introduction

Five stillbirths occur each minute, leading to an estimated global total of 1.9 million in 2021; 98% occur in low- and middle-income countries (LMICs) [1]. In sub-Saharan Africa and South Asia, third-trimester stillbirth rates are nearly tenfold higher than in high-income countries [2]. Despite a decline in global stillbirth rates over the past two decades, progress lags behind infant and child survival improvements, falling short of global targets [1]. In 2021, in sub-Saharan Africa and South Asia, the estimated stillbirth rate was 21.1 and 17 per 1000 total births, respectively [1]. The World Health Organization (WHO) Every Newborn Action Plan aimed to lower global stillbirth rates to 12 or fewer per 1,000 live births by 2030 [3]. To reach this goal, interventions must prioritize common causes of stillbirths, particularly in high-burden areas [1,3].

The dearth of quality data from LMICs on the etiology of stillbirths poses a significant challenge not only in constructing accurate statistics around the specific causes of fetal deaths but also in designing programs to prevent stillbirths. Our present understanding of stillbirths primarily stems from vital registration data and verbal autopsies [4]. However, both tools have substantial limitations, particularly when inferring etiologic causes. Many LMICs do not include stillbirths in vital registration systems, and verbal autopsies for stillbirths consist of only a few questions related to family reports of the events surrounding the stillbirth [4]. Neither approach involves a review of maternal medical records or a systematic biological investigation of the fetus and placenta, processes that are essential for determining causes of stillbirth [5]. A systematic review from 2018 showed that studies inconsistently applied classification systems for causes of stillbirth and suffered from poor-quality data, resulting in findings that were insufficient to guide prevention strategies [6]. In many countries, policies for identifying and documenting stillbirths are less developed compared to those for neonatal and under-five deaths. This gap likely contributes to the slow progress in reducing stillbirths, as inadequate data collection hinders targeted prevention and intervention efforts.

The Child Health and Mortality Prevention Surveillance (CHAMPS) Network aimed to track stillbirth and under-five mortality causes in South Asia and sub-Saharan Africa—regions with the highest stillbirth rates—using robust, standardized longitudinal data [7]. Early findings from the first two years of CHAMPS showed that intrauterine hypoxia was the main fetal cause of death, contributing to 72% of 180 stillbirths investigated using minimally invasive tissue sampling (MITS) [8]. Similar findings were reported in the PURPOSe study, which examined 611 stillbirths in India and Pakistan [9]; however, both studies had major limitations for informing global prevention strategies, either due to small sample sizes or limited geographic scope. In this paper we analyzed the placental, maternal, and fetal conditions leading to 2,492 stillbirths across seven CHAMPS countries in Sub-Saharan Africa and South Asia from 2016-2023, including identifying prevention opportunities missed.

## Methods

### Sites and mortality surveillance

A comprehensive description of CHAMPS sites and standardized mortality surveillance methods has been previously published [10]. Briefly, the CHAMPS Network consisted of sites in seven countries in sub-Saharan Africa and South Asia with defined catchment areas: Baliakandi and Faridpur, Bangladesh; Kersa, Harar, and Haramaya, Ethiopia; Bamako, Mali; Manhiça and Quelimane, Mozambique; Siaya and Kisumu, Kenya; Makeni and Bo, Sierra Leone; and Soweto, South Africa. Mozambique began enrollment in 2016, followed by South Africa, Mali, Kenya, and Bangladesh in 2017. Enrollment began in 2019 for Ethiopia and Sierra Leone.

CHAMPS defined stillbirths as babies delivered without spontaneous breathing or movement at delivery (and no response to resuscitation, if attempted), with a birth weight ≥1000 g or gestational age ≥ 28 weeks. Stillbirths were identified through facility- and community-based mortality surveillance systems within defined catchment areas at each site, where deaths were identified in near real-time by health facility staff or community informants. Site-specific mortality surveillance and death notification procedures were tailored to local capacity, cultural norms, and geography [11,12]. Upon notification of a death, surveillance teams verified eligibility and families were approached for consent. Stillbirths were eligible if they had occurred within the previous 24 hours or 36 hours if the body was refrigerated.

### Postmortem procedures and data collection

The complete methodology for MITS procedures is described in previous publications [13–15]. Briefly, following informed consent from guardians, trained teams conducted body inspections, anthropometry, and took photographs to assess dysmorphia and maceration. Biopsies of the lungs, brain, and liver, along with available placenta and umbilical cord, were collected using biopsy needles. To minimize contamination during MITS procedures, standardized aseptic techniques were followed across all sites. The stillborn body was disinfected with povidone-iodine prior to sampling, and single-use, sterile consumables were used. Personnel wore appropriate personal protective equipment, and dedicated procedure spaces were used where feasible. Specimen collection, processing, and laboratory analyses were conducted using standardized protocols, supported by regular training and quality assurance procedures. Tissue samples underwent histological examination at each site. Samples suggesting infection or diagnostic queries were further evaluated at the US Centers for Disease Control and Prevention. Blood, cerebrospinal fluid (CSF), lung tissue, and rectal swabs were also collected and tested for pathogens using polymerase chain reaction (PCR) on multiplexed TaqMan array cards (Thermo Fisher Scientific, Waltham, MA, USA); blood and CSF underwent conventional microbiological culture. Blood samples were also screened for tuberculosis, human immunodeficiency virus (via rapid tests/PCR), and malaria (through thick and thin smears and rapid tests). Clinical data from antenatal maternal health records and hospitalizations for delivery were abstracted, and trained teams interviewed caregivers using the 2016 WHO verbal autopsy instrument.

### Cause of death determination

A detailed description of the determination of cause of death (DeCoDe) process and diagnosis standards is available elsewhere [16]. In brief, all available data about each stillbirth, including maternal medical records, pathology reports, laboratory testing, photographs and other measurements, and verbal autopsy findings, were reviewed by local expert panels (DeCoDe panel) consisting of neonatologists, pediatricians, gynecologists, obstetricians, pathologists, microbiologists, and epidemiologists. Causes of death were recorded using the WHO International Classification of Diseases (ICD-10) and the WHO application of ICD-10 to deaths during the perinatal period (ICD-PM). Conditions in the causal chain were categorized into main and other fetal and maternal conditions [16].

Intrauterine hypoxia was assigned as the main fetal condition when supported by evidence consistent with an intrapartum event, such as placental pathology findings, documentation of reduced fetal movement or absent fetal heart rate in maternal records, meconium-stained amniotic fluid, cord prolapse, maternal cardiovascular collapse, or emergency cesarean delivery for fetal indications. Within the ICD-PM/ DeCoDe framework, maternal and obstetric conditions were assessed separately and recorded explicitly in the causal pathway rather than being incorporated into the fetal diagnosis. For fetal infectious conditions, the DeCoDe panel identified etiologic agents when possible. The panel also evaluated preventable factors, grouping findings for general prevention recommendations. In this study, preventability was assessed by DeCoDe panels within the local context of each site and refers to deaths that could potentially be avoided through optimal delivery and use of the care available in that setting at the time of death. Many of the DeCoDe panels provided detailed notes about how the stillbirth could have been prevented and we also presented granular prevention categories and subcategories based on detailed case-level recommendations when they were available. Detailed definitions of prevention subcategories are provided in Supplementary Table 1 in S1 File.

CHAMPS promoted comparability between DeCoDe panels through a network-wide quality assurance process designed to verify that panels across different sites applied diagnostic standards consistently and reproducibly. Each year, 5–6 cases per site were shared across the network for external review during routine panel meetings, and concordance was centrally scored and analyzed to identify areas where retraining, standard revisions, or process changes were needed. Refresher training was organized for each site every 6 months. In addition, CHAMPS convened in-person cross-network reviews of selected cases with panel representatives from each site and invited subject-matter experts to resolve challenging interpretations, strengthen causal-chain reasoning, and align cross-site practices annually, except during the peak of the COVID-19 pandemic due to travel restrictions.

### Statistical analysis

We used descriptive statistics to describe the characteristics of stillbirths and their causes and make comparisons between CHAMPS sites. We summarized categorical variables as frequencies with proportions and continuous variables as medians with interquartile ranges (IQR). Chi-squared test was used to test statistical differences between select groups of interest. Fisher’s exact test was used if the expected value was less than five for more than 20% of cells.

### Ethics statement

Written consent was obtained from the parents or guardians for postmortem procedures, including MITS, and data collection from medical records. All CHAMPS sites obtained approval from their respective ethics review committees to conduct the study. The CHAMPS Program Office, based at Emory University, received approval from the Emory University Institutional Review Board. In addition, since the initiation of CHAMPS, the Emory Center for Ethics has conducted ongoing reviews of program practices and policies. All CHAMPS protocols are available at https://champshealth.org/resources/protocols/.

## Results

From December 2016 through December 2023, CHAMPS teams approached parents and guardians of 4876 stillbirths eligible for MITS enrollment, 56% (2731/4876) provided consent and had MITS performed, with site-specific consent rates ranging from 32% (432/1356) in Bangladesh to 82% (323/394) in Kenya (**Supplementary Table 2, Supplementary Figure 1** in S1 File). Cause of death determination was completed for 2492 (91%) enrolled stillbirths, and these were included in the analysis; the remainder (n = 239) were still pending cause of death determination at the time of analysis. Nine percent (228/2492) of stillbirths were refrigerated prior to sampling; all others (91%) were sampled within 24 hours of delivery (Table 1). Overall, 51% (1282/2492) of stillbirths with MITS completed had no signs of maceration, and the proportion with no maceration varied significantly across sites, ranging from 27% (84/306) in South Africa to 61% (303/498) in Mozambique (Table 1). Sixty-three percent (1577/2492) weighed <2,500 g at the time of MITS, including 27% (671/2492) weighing <1500 g. Twenty one percent (526/2492) of stillbirths had an unknown gestational age, precluding our ability to discern if these babies were small-for-gestational age (Table 1). However, among the 69% (1726/2492) with information available on gestational age and MITS weight, 36% (625/1726) were small for gestational age according to the international newborn size for gestational age and sex INTERGROWTH-21st standards [17], again with high variation across sites, ranging from 21% (50/237) in Kenya to 52% (184/354) in Bangladesh (Supplementary Table 3). Seventy-five percent (1878/2492) of total stillbirths were vaginal deliveries, and 95% (2361/2492) were delivered at a healthcare facility (Table 1). No documentation on the presence of fetal heartbeat during labour was available in 55% (1367/2492) of cases (Table 1).

**Table 1:** Characteristics of 2492 stillbirths with cause of death determined, by CHAMPS site, 2016-2023.

Characteristics	All N=2492 n (%)	Bangladesh N=405 n (%)	Ethiopia N=568 n (%)	Kenya N=265 n (%)	Mali N=212 n (%)	Mozambique N=498 n (%)	Sierra Leone N=238 n (%)	South Africa N=306 n (%)
Stillbirth type								
Fresh	1282 (51)	165 (41)	339 (60)	127 (48)	126 (59)	303 (61)	138 (58)	84 (27)
Macerated	1120 (45)	237 (59)	219 (39)	120 (45)	83 (39)	171 (34)	94 (39)	196 (64)
Unknown	90 (4)	3 (1)	10 (2)	18 (7)	3 (1)	24 (5)	6 (3)	26 (8)
Stillbirth timing								
Antepartum	1077 (43)	227 (56)	171 (30)	77 (29)	91 (43)	190 (38)	105 (44)	216 (71)
Intrapartum	825 (33)	130 (32)	231 (41)	102 (38)	41 (19)	120 (24)	128 (54)	73 (24)
Unknown	590 (24)	48 (12)	166 (29)	86 (32)	80 (38)	188 (38)	5 (2)	17 (6)
Sex								
Male	1330 (53)	206 (51)	290 (51)	144 (54)	113 (53)	270 (54)	129 (54)	178 (58)
Female	1154 (46)	199 (49)	277 (49)	121 (46)	99 (47)	224 (45)	109 (46)	125 (41)
Unknown	8 (0)	0 (0)	1 (0)	0 (0)	0 (0)	4 (1)	0 (0)	3 (1)
Gestational age, weeks								
Preterm	1136 (46)	261 (64)	158 (28)	140 (53)	81 (38)	208 (42)	111 (47)	177 (58)
Term	777 (31)	125 (31)	128 (23)	100 (38)	69 (33)	175 (35)	101 (42)	79 (26)
Postdates	53 (2)	11 (3)	0 (0)	12 (5)	4 (2)	21 (4)	2 (1)	3 (1)
Unknown	526 (21)	8 (2)	282 (50)	13 (5)	58 (27)	94 (19)	24 (10)	47 (15)
Fetal heartbeat during labor								
Present	342 (14)	12 (3)	110 (19)	69 (26)	10 (5)	85 (17)	34 (14)	22 (7)
Absent	783 (31)	58 (14)	222 (39)	116 (44)	67 (31)	114 (23)	106 (45)	100 (33)
Unknown	1367 (55)	335 (83)	236 (42)	80 (30)	135 (64)	299 (60)	98 (41)	184 (60)
Weight at time of MITS, grams								
<1,000	177 (7)	41 (10)	57 (10)	15 (6)	20 (9)	7 (1)	12 (5)	25 (8)
1,000-1,499	494 (20)	115 (28)	157 (28)	22 (8)	41 (19)	48 (10)	25 (11)	86 (28)
1,500-2,499	906 (36)	177 (44)	181 (32)	96 (36)	73 (34)	184 (37)	77 (32)	118 (39)
2,500-3,999	845 (34)	66 (16)	164 (29)	119 (45)	74 (35)	237 (48)	115 (48)	70 (23)
4,000-5,400	59 (2)	4 (1)	9 (2)	13 (5)	4 (2)	19 (4)	9 (4)	1 (0)
Unknown	11 (0)	2 (0)	0 (0)	0 (0)	0 (0)	3 (1)	0 (0)	6 (2)
*Small-for-gestational age								
Yes	625 (25)	184 (45)	61 (11)	50 (19)	56 (26)	121 (24)	57 (24)	96 (31)
No	1101 (44)	170 (42)	117 (21)	187 (71)	85 (40)	242 (49)	155 (65)	145 (47)
Unknown	766 (31)	51 (13)	390 (69)	28 (11)	71 (33)	135 (27)	26 (11)	65 (21)
Type of delivery								
Vaginal delivery	1878 (75)	322 (80)	513 (90)	204 (77)	144 (68)	313 (63)	160 (67)	222 (73)
Caesarean section	563 (23)	82 (20)	49 (9)	61 (23)	45 (21)	172 (35)	77 (32)	77 (25)
Assisted delivery	13 (1)	1 (0)	4 (1)	0 (0)	4 (2)	2 (0)	0 (0)	2 (1)
Unknown	38 (2)	0 (0)	2 (0)	0 (0)	19 (9)	11 (2)	1 (0)	5 (2)
Type of gestation								
Singleton	2286 (92)	380 (94)	525 (92)	247 (93)	184 (87)	439 (88)	222 (93)	289 (94)
Multiple	206 (8)	25 (6)	43 (8)	18 (7)	28 (13)	59 (12)	16 (7)	17 (6)
Maternal age, years								
Unknown	558 (22)	27 (7)	178 (31)	31 (12)	39 (18)	139 (28)	13 (5)	131 (43)
≤19	266 (11)	67 (17)	26 (5)	26 (10)	23 (11)	75 (15)	31 (13)	18 (6)
20-24	519 (21)	104 (26)	110 (19)	70 (26)	35 (17)	101 (20)	66 (28)	33 (11)
25-29	511 (21)	88 (22)	133 (23)	61 (23)	48 (23)	74 (15)	64 (27)	43 (14)
30-34	376 (15)	71 (18)	72 (13)	46 (17)	35 (17)	68 (14)	42 (18)	42 (14)
≥35	262 (11)	48 (12)	49 (9)	31 (12)	32 (15)	41 (8)	22 (9)	39 (13)
Place of delivery								
At health facility	2361 (95)	399 (99)	532 (94)	258 (97)	186 (88)	469 (94)	232 (97)	285 (93)
Outside facility	89 (4)	6 (1)	34 (6)	7 (3)	7 (3)	15 (3)	3 (1)	17 (6)
Unknown	42 (2)	0 (0)	2 (0)	0 (0)	19 (9)	14 (3)	3 (1)	4 (1)
Placenta Collection								
Placenta collected	1224 (49)	303 (75)	160 (28)	115 (43)	64 (30)	326 (65)	96 (40)	160 (52)
Placenta not collected	1268 (51)	102 (25)	408 (72)	150 (57)	148 (70)	172 (35)	142 (60)	146 (48)
Time from delivery to MITS procedure, hours								
≥24	228 (9)	0 (0)	4 (1)	36 (14)	2 (1)	41 (8)	3 (1)	142 (46)
<24	2264 (91)	405 (100)	564 (99)	229 (86)	210 (99)	457 (92)	235 (99)	164 (54)
Median (IQR)	7 (2-16)	1 (1-2)	3 (1-4)	16 (10-21)	9 (4-15)	14 (7-19)	9 (4-13)	24 (13-33)

*Gestational age was not usually assessed by an early ultrasound.

** n(%): Percentages calculated using total country-level N as the denominator; due to rounding, percentages may not always sum to 100%.

### Conditions in the fetus leading to death

DeCoDe panels were able to determine a cause of death (fetal or maternal condition) for 94% (2342/2492) of stillbirths reviewed. Among those with a cause determined, one main condition in the fetus leading to death was identified in 97% (2278/2342) (**Supplementary Table 4** in S1 File); 64 stillbirths (3%) had other fetal conditions in addition to the main condition in the causal chain. Intrauterine hypoxia, defined as the hypoxic condition of a fetus starting before or during labor, was determined to be the main condition in the fetus in 75% (1864/2492) of all stillbirths and was consistently the predominant main condition regardless of site, ranging from 59% (182/306) in South Africa to 89% (236/265) in Kenya (Fig 1**, Supplementary Table 4** in S1 File). Among cases where intrauterine hypoxia was identified as the main fetal condition, 18% (322/1864) had no identifiable maternal or placental condition (**Supplementary Table 6** in S1 File). In these cases, intrauterine hypoxia was assigned as the main fetal condition based on histopathological evidence from lung tissue, even in the absence of documented maternal or placental abnormalities. Congenital birth defects accounted for 9% (218/2492) of main conditions in the fetus, ranging from 2% (4/265) in Kenya to 24% (134/568) in Ethiopia (Fig 1, **Supplementary Table 4** in S1 File). Sixty-two percent (135/218) of congenital birth defects were neural tube defects, most of which (82%, 110/134) occurred in the Ethiopia site. Congenital infection was the third most common condition in the fetus for 8% (200/2492) of all stillbirths, ranging from 1% (3/238) in Sierra Leone to 28% (87/306) in South Africa (**Supplementary Table 4** in S1 File).

**Fig 1:**
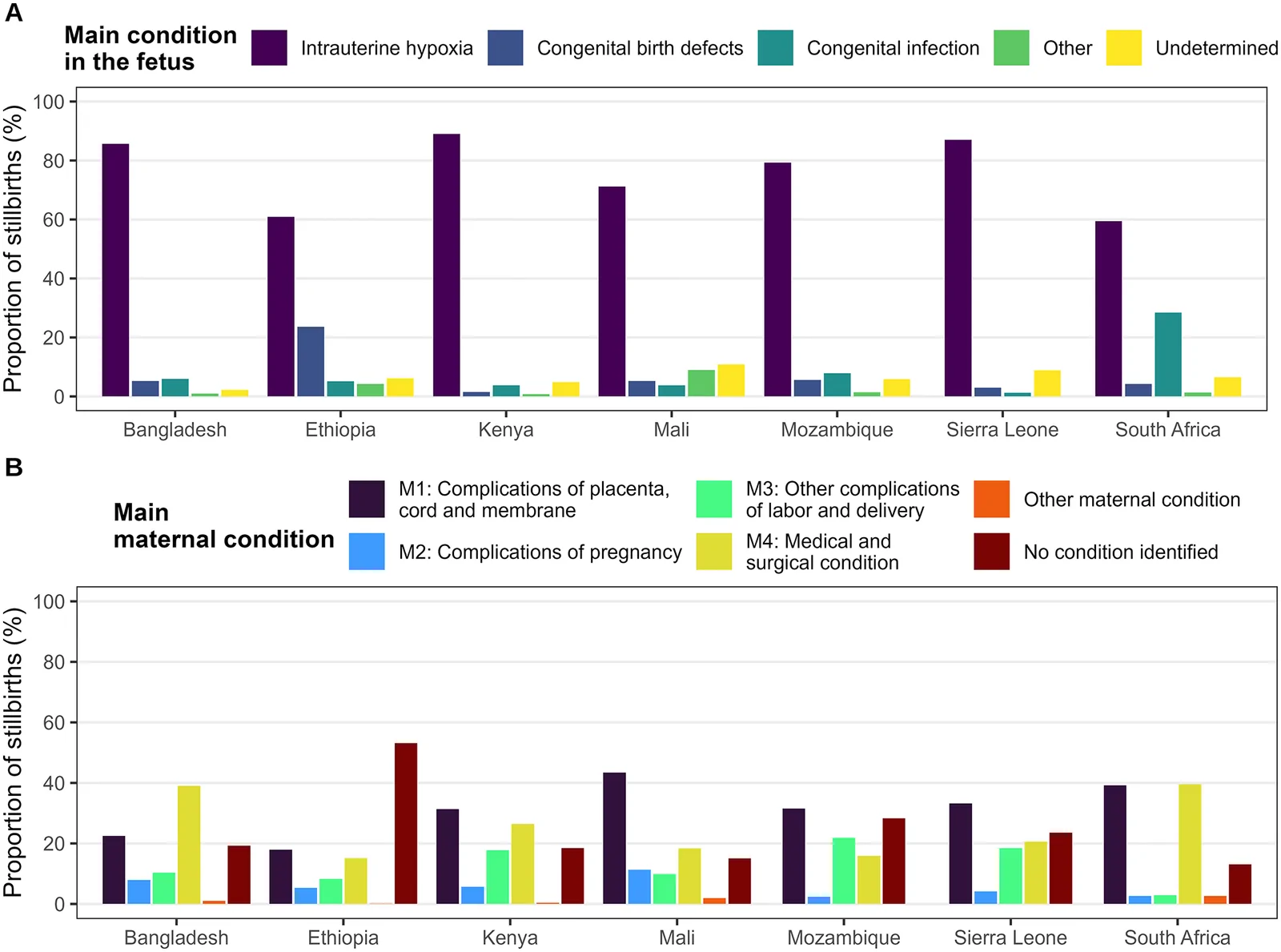
Causes of death among 2492 stillbirths, by CHAMPS site, 2016-2023, A) Main condition in the fetus, and B) Main maternal condition.

### Etiologies of congenital infections

A total of 245 etiologic agents were identified in the causal chain among the 228 stillbirths with congenital infection (**Supplementary Table 4** in S1 File). Sixty-six percent had one etiologic agent detected (150/228), 12% (27/228) had two, 5% (11/228) had three, and 1% (2/228) had four. Forty-five percent of all stillbirths with congenital infection in the causal chain had one or more gram-positive bacteria (102/228), 51% (116/228) had one or more gram-negative bacteria, 10% (23/228) had one virus, 1% (2/228) had one protozoa and 1% (1/228) had one fungus (**Supplementary Table 4, Supplementary Table 7** in S1 File).

The primary infectious causes of stillbirths were Group B Streptococcus (2%, 46/2492), *Escherichia coli* (2%,43/2492), and *Enterococcus faecalis* (1%, 24/2492) (**Supplementary Table 7** in S1 File). Almost all viral infections were due to cytomegalovirus, contributing to 1% of all stillbirths (20/2492). Congenital syphilis was only reported as a cause of stillbirths in South Africa (5%, 14/306), Mozambique (1%, 4/498), and Bangladesh (0.5%, 2/405) (**Supplementary Table 7** in S1 File).

### Comparison of conditions in antepartum and intrapartum stillbirths

Seventy-six percent (1902/2492) of stillbirths had information available to determine whether the death occurred antepartum or intrapartum (**Table 1, Supplementary Table 8** in S1 File). The distribution of the main condition in the fetus across the antepartum, intrapartum, and stillbirths with unknown timing was similar (**Supplementary Table 9** in S1 File). Most stillbirths with an undetermined condition in the fetus were antepartum stillbirths (53%, 79/150). Macerated stillbirths accounted for a higher proportion of antepartum cases (67%, 724/1077) compared to stillbirths without maceration (30%, 322/1077) (**Supplementary Table 9** in S1 File).

### Main maternal condition contributing to fetal death

We identified a maternal condition contributing to fetal death in 72% (1794/2492) of stillbirths (**Supplementary Table 4** in S1 File). In 5% (122/2492) of cases, maternal conditions remained unknown primarily due to missing data in maternal medical records. Additionally, in 23% (576/2492), specific maternal conditions could not be identified even though maternal medical records were available; poor quality and completeness of records contributed to many of these cases (**Supplementary Table 10** in S1 File). The most common group of maternal conditions based on ICD-PM classifications was complications of the placenta, cord, and membranes (29%, 724/2492) (Fig 1, Supplementary Table 4). The second most common group was medical and surgical conditions (24%, 602/2492) (Fig 1, Supplementary Table 4), largely consisting of maternal hypertension (17%, 414/2492) (**Supplementary Table 6** in S1 File).

Forty-nine percent (1224/2492) of stillbirths had placenta collected, with significant variation across sites, ranging from 28% (160/568) in Ethiopia to 75% (303/405) in Bangladesh (Table 1), and 37% (458/1224) of stillbirths with a placenta examined were attributed to placenta, membrane, and cord related complications (**Supplementary Table 5** in S1 File). Among the 1268 stillbirths where placenta was not available, placenta-related complications (placental, membrane, and cord complications) were determined based only on maternal clinical records and verbal autopsy interviews and were less frequently identified than among stillbirths with placenta available for investigation (21%, 266/1268 vs 458/1224) (**Supplementary Table 5** in S1 File). There was no correlation between the collection and investigation of the placenta for stillbirths and the proportion of stillbirths where a main maternal condition was identified across sites. (**Supplementary Figure 2** in S1 File) Placental complications (22%, 415/1864) and maternal hypertension (20%, 382/1864) were the primary conditions among the subset of stillbirths with intrauterine hypoxia as the main condition in the fetus (**Supplementary Table 6** in S1 File**).** Chorioamnionitis and membrane complications (36%, 73/200) and maternal infection (12%, 25/200) were the primary maternal conditions among stillbirths with congenital infections. We identified only 9 stillbirths where the maternal infectious cause was HIV infection (**Supplementary Table 6** in S1 File), reported from three countries: Kenya (5/265), Sierra Leone (2/238), and South Africa (2/306). No maternal condition was identified for 88% (191/218) of stillbirths with congenital birth defects as the main fetal condition. Among the 69% of stillbirths (1726/2492) for whom small-for-gestational-age was able to be determined, 36% (625/1726) were classified as small-for-gestational-age. Among these stillbirths, the most frequently identified contributing conditions were maternal hypertension (23%, 141/625) and placental complications (21%, 129/625; **Supplementary Table 11** in S1 File).

Stratification by gestational age (1966/2492) showed that intrauterine hypoxia remained the predominant fetal condition across all groups (75% preterm, 81% term, 81% postdate). Maternal conditions varied by gestational age, with hypertension and placental complications more common in preterm stillbirths (22% each), while intrapartum-related conditions such as obstructed labor were more frequent in term stillbirths (11%) (**Supplementary table 12** in S1 File).

### Prevention

Seventy-two percent (1808/2492) of stillbirths were considered preventable by DeCoDe panels, under optimal provision of existing care (Table 2). Improving antenatal care and obstetric care and management was the most frequently recommended action to prevent stillbirths across all sites, among the 85% (1532/1808) of stillbirths considered preventable (**Supplementary Table 13** in S1 File). A further 27% could have been prevented by improved health-seeking behavior on the part of families (491/1808), with the highest proportion reported in Ethiopia (57%, 267/470). Recommendations also emphasized improving family planning in Ethiopia (53%, 247/470) and prioritized HIV prevention and control in Kenya (24%, 60/250) (**Supplementary Table 13** in S1 File).

**Table 2:** Preventability of 2492 stillbirths with complete cause of death determination, by main condition in the fetus, 2016-2023.

	Total N = 2492 n (%)	Intrauterine hypoxia N = 1864 n (%)	Congenital birth defect N = 218 n (%)	Congenital infection N = 200 n (%)	Intrauterine growth restriction N = 32 n (%)	Other condition in fetus N = 28 n (%)	Only maternal condition N = 39 n (%)	No condition in the fetus nor in mother identified N = 111 n (%)
Preventable given current situation	1531 (61)	1257 (67)	112 (51)	105 (52)	22 (69)	16 (57)	19 (49)	0 (0)
Preventable given ideal situation	277 (11)	232 (12)	13 (6)	10 (5)	6 (19)	3 (11)	13 (33)	0 (0)
Not preventable	499 (20)	322 (17)	78 (36)	80 (40)	3 (9)	9 (32)	7 (18)	0 (0)
Not possible to determine with current data	185 (7)	53 (3)	15 (7)	5 (2)	1 (3)	0 (0)	0 (0)	111 (100)

Among 1099 (44%) preventable stillbirths where the site DeCoDe panel provided more detailed information on prevention recommendations, we were able to categorize them into more specific prevention measures, sixty nine percent (753/1099) of those stillbirths could have been prevented through improved antenatal care, which includes quality antenatal care, management of medical conditions and health education as the three most common subcategories (Fig 2). Thirty-three percent of stillbirths could have been prevented through improved obstetric management (361/1099), ranging from 25% in Mozambique (11/44) and South Africa (10/40) to 36% in Mali (38/106) and Kenya (81/227), and 10% through delivery by a qualified provider at a healthcare facility (105/1099), mostly in Sierra Leone (15%, 17/110) and Kenya (14%, 31/227). In addition, 26% could have been saved through improved documentation of medical records in Kenya (Fig 2). Forty percent of congenital infections were not considered preventable (Table 2), primarily because there are currently no maternal screening guidelines or interventions for the causative pathogens.

**Fig 2:**
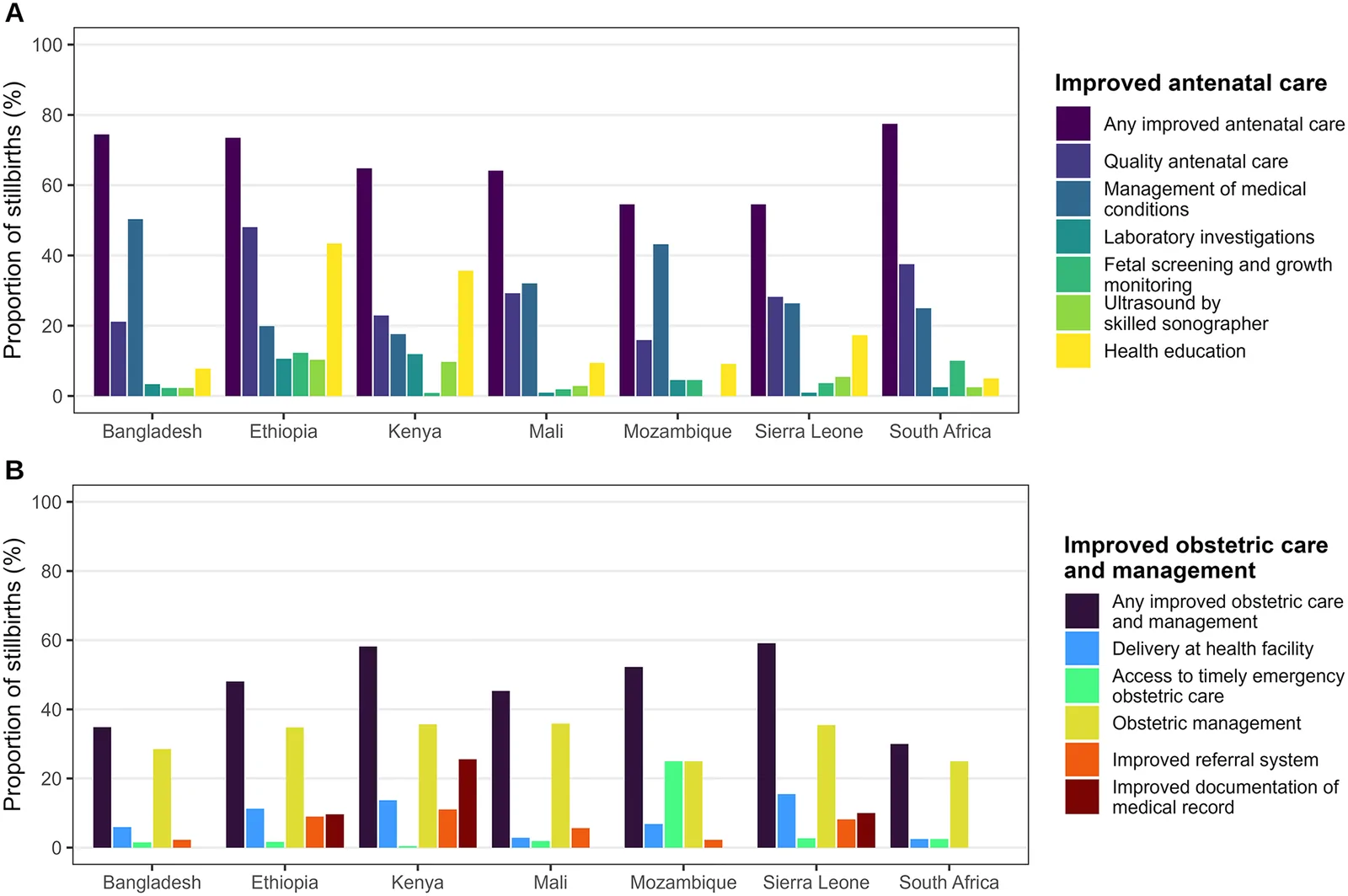
Detailed recommendations regarding antenatal care and obstetric care and management that could have prevented stillbirths, among those deemed preventable with details provided by DeCoDe panels, by CHAMPS site, 2016-2023, N = 1099.

## Discussion

Our investigation of 2492 stillbirths across seven years in seven countries consistently identified intrauterine hypoxia linked to maternal conditions like hypertension or placenta or cord abnormalities as the most common causes across CHAMPS sites, with congenital infections, and birth defects as leading causes when a condition was identified in the fetus. Our findings support earlier findings from CHAMPS and the South Asia specific PURPOSe study [9]. Because 95% of stillbirths in this analysis occurred in health facilities, these findings are particularly informative for identifying gaps across the health system, including antenatal, referral, and facility-based care, for guiding targeted interventions. Seventy-two percent (1808/2492) of stillbirths were considered preventable by DeCoDe panel and most could have been prevented through early detection and management of maternal hypertension and placental dysfunction. Improving the quality of antenatal and intrapartum care, ensuring timely access to emergency obstetric services, and implementing targeted infection screening and prevention are also critical—particularly in settings where infectious causes are more prevalent.

However, we identified important geographic differences in the causes of stillbirths, likely due to heterogeneity in underlying epidemiologic patterns and access to quality antenatal and obstetric care. These differences highlight the need to tailor prevention strategies to the specific healthcare context of each country. For example, at the preconception and early antenatal stage, systemic folate fortification in Ethiopia will be key to reducing neural tube defects, including both population level fortification and periconceptional folic acid supplementation. This cause of death was uncommon in other sites, all of which have folate-fortified foods [18]. In Mozambique, Mali, and Bangladesh, the main strategy for preventing stillbirths was through improving access to quality antenatal care to adequately manage maternal medical conditions, such as hypertension. In contrast, in Ethiopia, Sierra Leone, and South Africa, access to antenatal care was less of a concern, but the quality of care was more important. We also observed substantial geographic heterogeneity in stillbirth from congenital infections; nearly a third of all stillbirths in Soweto, South Africa, were attributed to congenital infections compared to 2–10% at other sites.

Approximately three-quarters of stillbirths had intrauterine hypoxia as the main fetal condition. Despite the numerous differences between CHAMPS sites and contexts, this pattern was consistent. This diagnosis implies that no specific abnormality or disease was identified in the fetus but that pregnancy or delivery complications led to stillbirth. The two most common maternal conditions identified among stillbirths with intrauterine hypoxia were maternal hypertension and placental complications. Over the past two decades, pregnancy-related hypertensive disorders and broadly adult hypertension have remained highly prevalent in sub-Saharan Africa and South Asia [19,20]. Globally, the proportion of pregnancies affected by hypertensive disorders increased by 11% between 1990–2019 (from 16.30 million to 18.08 million cases). Although the age-standardized incidence rate per 100,000 population decreased from 579 to 463 over the same period [20]. Nevertheless, pregnant women experience a series of barriers in diagnosing and managing hypertension and, ultimately, in preventing stillbirths. In South Africa, clinical evidence from a seven-year population-based study revealed that 40% of mothers with hypertensive disease presented only after the condition had escalated to life-threatening states, such as severe pre-eclampsia, eclampsia, or HELLP syndrome [21]. This delayed presentation suggests that hypertensive status frequently remains undetected during the earlier, manageable stages of pregnancy. This delay in diagnosis is often made worse by a lack of awareness among mothers. For example, 41.8% of pregnant women in Ethiopia lacked awareness of pregnancy-induced hypertension as a potential risk during pregnancy [22]. These challenges are compounded by inadequate access or sociocultural barriers to antenatal care [23] and poor screening practices when antenatal care is sought. For instance, observational data from similar low resource settings indicate that only 31% women received the full package of WHO-recommended screening (such as both blood pressure and proteinuria checks) during their antenatal visits [24,25]. Across multiple sites, where intrauterine hypoxia linked to maternal hypertension and placental complications was predominant, strengthening antenatal screening, timely diagnosis, and management of hypertensive disorders and placental dysfunction is critical [26]. Existing interventions have shown significant potential in these contexts; for instance, the implementation of third-trimester Doppler ultrasound (e.g., Umbiflow) in primary care settings has been associated with a reduction in stillbirth rates by up to 38% [27,28]. Furthermore, initiating low-dose aspirin prophylaxis between 11–14 weeks for high-risk women and utilizing biomarkers such as placental growth factor (PlGF) can facilitate earlier recognition of placental insufficiency and pre-eclampsia [29,30]. Other complications, such as placental abruptions, are often unpredictable and remain challenging to prevent with current medical technologies [31]. Although established clinical guidelines provide a framework for management based on maternal and fetal compromise and gestational age, the effectiveness ultimately depends on rapid access to emergency obstetric care [32]. Interventions such as maternity waiting homes to reduce travel distance, community-based emergency transport schemes, and digital coordinated referral systems (e.g., m-mama) may help reduce delays in reaching care [33–35].

In settings where intrapartum-related deaths remain substantial, improving the quality and timeliness of intrapartum care—including fetal monitoring and access to emergency obstetric services—should be prioritized [4,36,37]. Clinical guidelines are only effective if a mother can reach a healthcare facility in time. Systems that ensure rapid access to emergency surgical and resuscitative capacity are essential, because delays in reaching care are directly associated with adverse maternal and fetal outcomes [38]. In Mozambique, access to timely and appropriate obstetric care was a major gap in prevention highlighting the need to strengthen referral linkages, emergency transport systems, intrapartum monitoring, and facility readiness for rapid obstetric management [39].

In contrast, in sites where congenital infections contributed a larger share of stillbirths, such as South Africa, targeted maternal infection screening, prevention, and treatment strategies during pregnancy may have greater impact. The most common pathogens causing congenital infections, including Group B Streptococcus, *Escherichia coli*, and Enterococcus faecalis, are pathogens commonly associated with rectovaginal tract colonization in pregnant women, as well as invasive infections among stillbirths in Soweto [40,41]. Although syphilis screening programs already exist, the rising burden of syphilis suggests the need to strengthen repeat testing protocols, such as rapid screening every four weeks to align with existing HIV testing schedules [42]. In addition, given the significant burden of Group B Streptococcus (GBS) identified in these settings, the development and implementation of maternal GBS vaccines represent a high-impact strategy for preventing infection-related stillbirths [21].

Among the 69% of stillbirths (1726/2492) with sufficient information on gestational age, sex, and birthweight to determine small-for-gestational-age (SGA), 36% were classified as SGA. Previous study have classified small-for-gestational-age stillbirths as part of the small vulnerable newborns group, but they have not been fully integrated into burden assessments [43]. In our study, the majority of SGA-related stillbirths were attributed to maternal hypertension and placental complications. However, because SGA status could not be determined for nearly one-third of stillbirths, its contribution may be underestimated. More complete gestational age assessment improved fetal growth monitoring during pregnancy, and better documentation of fetal status at delivery would likely improve identification of growth-restricted fetuses and improve understanding of their contribution to stillbirth [44].

Ninety-five percent of stillbirths in this study occurred in facility settings, which is similar to the proportion of all deliveries in South Africa (96%) and Kenya (88%) but far higher than national facility delivery rates in countries such as Ethiopia (48%) and Bangladesh (65%) [45]. MITS depends on rapid death identification—typically within 24 hours—and stillbirths occurring in the community are less likely to be identified quickly and enrolled. The events leading up to a stillbirth are as story about the events befalling a mother; however, we were frequently limited by missing information about mothers. Incomplete maternal records, including missing gestational age (21%), timing of fetal death (24%) and discarded placentas (51%), limited our ability to understand the causal chain leading to death. In particular, the lack of fetal heartbeat monitoring during delivery reduced our ability to distinguish between antepartum and intrapartum deaths, which in turn limited precise identification of prevention opportunities. Placental collection also remained a major challenge. Placentas were available for only half of enrolled stillbirths, largely because they were not routinely preserved in health facilities and were often discarded before consent could be obtained. Although collected placentas underwent gross and histological examination, some infectious causes may have gone undetected because placental molecular testing was not routinely performed. For example, the PURPOSe study detected *Ureaplasma urealyticum* in 39% of placentas they studied using molecular test methods, although detection of a pathogen alone does not necessarily mean that the infection would be assigned in the causal chain. More broadly, incomplete placental and maternal infectious disease evaluation in our study may have led to under ascertainment of infectious contributors to stillbirth. This is particularly relevant for maternal infections, which may be missed without targeted testing, as illustrated by recent evidence from Bangladesh identifying scrub typhus caused by *Orientia tsutsugamushi* as a likely contributor to stillbirths and neonatal deaths [46]. Our current understanding of some maternal conditions is primarily based on maternal medical records and verbal autopsy, which were frequently unavailable or incomplete, either because tests were not conducted or the results were not recorded. A comprehensive investigation into the mother’s health, including recommended laboratory testing and fetal monitoring, would enhance our understanding of the causal chain and opportunities for prevention, and would also likely improve outcomes, as these form part of quality antenatal and obstetric care. Performing routine karyotyping or, preferably, chromosomal microarray analysis on the stillbirths in this study would likely have influenced the distribution of proportions assigned to various causes and would be especially valuable in analyses of stillbirths with congenital anomalies [47].

CHAMPS is the longest-running systematic postmortem investigation into causes of stillbirths, and our findings are representative of stillbirths occurring at each site, particularly those occurring in healthcare facilities [18]. This analysis solidifies our prior understanding of the causes of stillbirths and identifies some geographic heterogeneity in the proportion of stillbirths linked to major causes. Furthermore, it highlights the myriad persistent gaps in access to quality maternal and obstetric care, and subsequent gaps in information about the stillbirth, that must be urgently addressed to reduce stillbirths and better define their underlying causes.

### Data sharing

CHAMPS data are available online and requests for further detailed deidentified data for research and evaluation purposes can be made at: https://champshealth.org/data/.

## Supporting information

S1 File Supplementary Tables and Figures. (ZIP)
