# Supplementary material for: Causes of stillbirth in sub-Saharan Africa and South Asia: Findings from Child Health and Mortality Prevention Surveillance, 2016–2023
Source: PLOS Glob Public Health. 2026 Oct 27;6(10):e0005235. doi: 10.1371/journal.pgph.0005235 (PMC13712675; doi:10.1371/journal.pgph.0005235)
Supplement: Supplementary file 2 [file pgph.0005235.s003.docx]

17 May 2026

Dear PLOS Global Health Editor,

We sincerely appreciate the opportunity to respond to the reviewers’ thoughtful comments and suggestion for our paper. Please find attached our point-by-point responses to these suggestions, along with a revised manuscript with the changes tracked for easy identification. We believe that the paper has improved with these revisions, and we hope you agree.

Best regards,

Emily Gurley

Corresponding author

**Response to Editorial Comments (Reviewer 1)**

- Although it is clear that undertaking this kind of review is demanding, the diversity in healthcare quality, variability in disease prevalence across the two study regions, and differences in cultural practices suggest that the study outcomes could have been more generalizable if additional sites had been included. The review, however, did not sufficiently address this limitation.

For instance, based on 2015 estimates, Nigeria, one of the most populous nations in the world, contributed approximately 12.2% of global stillbirths. Furthermore, the 2024 UNICEF/UN Inter-Agency Report indicates that Nigeria recorded about 184,000 stillbirths in 2023, representing roughly 20% of all stillbirths in Sub-Saharan Africa (approximately 912,000). Given Nigeria’s substantial contribution to both the regional and global stillbirth burden, the complete absence of data from the country places a significant constraint on the generalisability of the findings.

It should therefore be explicitly stated that the results must be interpreted with caution, acknowledging this as a key limitation of the study.

**Response:** CHAMPS sites have been selected to represent a wide variety of contexts across Africa and South Asia, where burden of mortality is the highest. However, we agree with the comment that CHAMPS does not to provide representative data from across all these regions. In fact, the sites selected for CHAMPS are not even representative within the countries they represent as the specific sites were chosen to reflect relatively high mortality areas. Thus, our interpretation of findings from CHAMPS is that when we find similar patterns across all CHAMPS sites, despite their major differences, we can have more confidence that these findings are likely generalizable across both regions. However, when we observe differences, these could be due to regional differences or differences within those specific CHAMPS catchment areas. Our discussion section (second paragraph of the limitation section) cautions the reader about generalization of our findings.

- It would be informative if the authors could clarify the proportion of stillbirth data obtained from community settings versus hospital-based facilities across the various study sites. This distinction is crucial for understanding potential selection bias and assessing the representativeness of the study population.

**Response:** We agree that the source of stillbirth identification is relevant and have now clarified this in the manuscript. Overall, the majority of stillbirths (95%) were delivered in health facilities, although this varied modestly across sites. In the discussion section (paragraph 7), we also compare this proportion to the proportion of all births from our sites that occur in facilities to provide context regarding representativeness.

- In addition, the authors are encouraged to present the key findings using infographics or other visual data summaries to enhance readability, facilitate comparative interpretation, and improve the overall clarity of data presentation.

**Response:** We have presented key findings from the paper in figures that we hope are easy for the reader to understand. If the reviewer has specific suggestions for findings that might be best presented in another visual format, we would be happy to receive the suggestion.

- Could the authors project the estimated year when the global stillbirth reduction target might be achieved based on current trends? Including this analysis, even briefly in the abstract, would underscore the urgency for action and highlight the pace of progress

**Response:** We agree with the reviewer that these projections would be useful for public health action. However, CHAMPS catchment areas were selected to represent high mortality areas to drive interventions. Since we are not representative of our countries or regions, we do not have the data to allow us to perform these projections.

- The Methods section is too brief and lacks sufficient detail for replication. Not all readers will have access to the referenced studies in the methods section. It would be helpful to provide a concise summary of the surveillance framework within the manuscript or include a graphical representation of the surveillance system to aid understanding. Describe the notification and reporting process used in the surveillance system.

**Response:** We have revised the methods section and added key details in the second paragraph of the method as suggested by the reviewer.

- Considering the wide variability in consent rates for MITS across study sites, there is a potential for differential data quality and possible misrepresentation of the true distribution of causes of death. To strengthen the validity of the findings, the authors are encouraged to apply appropriate statistical weighting or conduct sensitivity analyses when combining data from sites with markedly different consent proportions. This will help ensure more robust and generalisable conclusions.

**Response:** Consent rates indeed varied across study sites. For these differences to lead to misrepresentations of cause of stillbirth, the cause of the death would have to be related to the probability of consent. However, we are not aware of evidence suggesting that there is any relationship between cause of death and likelihood of consent, rather this is primarily driven by cultural sensitivities. Our discussion section cautions the reader about overgeneralizing results from our study.

- The authors should recheck the results to ensure that the appropriate denominators were used in all calculations and data presentations

**Response:** We have rechecked all denominators in the paper and made the necessary edits where applicable.

- What proportion of stillbirths were refrigerated prior to sampling?

**Response:** 9% of stillbirths were refrigerated prior to sampling; all others were sampled within 24 hours of delivery. This has been included in the result section paragraph 1.

- What was the mean or median time from stillbirth to specimen collection at each site?

**Response:** The median time from delivery to sampling across sites was 7 hours (IQR: 2–16), with site-specific medians ranging from 1 hour to 24 hours; all stillbirths where MITS was performed >24 hours after delivery were refrigerated to slow decomposition. These data are presented in Table 1.

- The manuscript should briefly discuss any laboratory or logistical constraints, including instances of delayed sample collection, autolysis, or bacterial overgrowth that might affect the accuracy of MITS findings.

**Response:** We followed standardized, network-wide protocols for sample collection, including refrigeration when collection was delayed beyond 24 hours. We have mentioned about this in the second paragraph of the method section.

- The manuscript contains too many self-citations. The authors should include additional relevant references from independent sources to ensure balance and enhance the credibility of the work.

**Response:** Self-citations were primarily included in the Methods section to reference previously published CHAMPS protocols and standardized procedures, ensuring methodological transparency and reproducibility. In this revision, we have added relevant references to the discussion.

**Response to Editorial Comments (Reviewer 2)**

- The paper reports that 56% of eligible families consented to MITS, with wide site variation (e.g., 32% Bangladesh vs 82% Kenya). This raises concerns about selection bias and comparability across sites. Provide a comparison of MITS-consented vs non-consented eligible stillbirths (where available): maternal age, location (facility/community), maceration status, gestational age/weight, timing since death notification, etc. and explicitly discuss how consent variation could influence the observed distribution of causes (e.g., facility vs community deaths, differences in availability of clinical records/placenta)

**Response:** We agree that variation in MITS consent rates across sites could affect comparability and should be acknowledged clearly. Our ability to compare MITS-consented and non-consented eligible stillbirths is limited because detailed information is not consistently available when families do not consent. We have therefore addressed this concern by clarifying this limitation in the manuscript and by discussing how differences in death location and in the availability of information across sites may influence the observed distribution of causes. We have added it as a limitation in the discussion (paragraph seven of Discussion section).

We do not suggest that these findings are representative of all stillbirths in the country. Rather, we interpret them as reflecting stillbirths identified within the CHAMPS surveillance populations in the facilities and communities where surveillance was conducted. In the revised Discussion, we now note that variation in consent rates across sites, together with differences in the proportion of facility and community deaths, which may influence the observed distribution of causes. At the same time, several findings remained consistent across sites despite these differences, which supports the relevance of those patterns across diverse settings.

- “Intrauterine hypoxia” is assigned as the main fetal condition in 75% overall, and remains dominant across sites. Because this category can function as a “final common pathway,” readers will ask how often it is a specific diagnosis versus a label used when no fetal pathology is identified. Provide a clear operational definition in the main text (not only referenced standards): how panels distinguish hypoxia due to intrapartum events, maternal hypertension, placental abruption, cord events, etc. and Quantify how often hypoxia is assigned with identified upstream maternal/placental conditions vs unexplained (you mention 18% without underlying maternal/placental causes bring this into the main Results narrative with a short interpretation).

**Response:** This diagnosis was not used as a nonspecific label when no fetal pathology was identified. Rather, panels assigned the diagnosis based on the strength of available evidence. For intrauterine hypoxia specifically, evidence supporting intrapartum onset were all relevant for assigning cause, including placental pathology consistent with an intrapartum event, maternal report of reduced fetal movement or absent fetal heart rate from the maternal medical record, meconium-stained amniotic fluid, cord prolapse, maternal cardiovascular collapse, or emergency cesarean delivery for fetal indications. We have further clarified that maternal and obstetric conditions are considered separately within the ICD-PM/DeCoDe framework rather than being absorbed into the fetal diagnosis. Accordingly, conditions such as maternal hypertension, placental abruption, and cord complications are assessed explicitly and recorded separately in the causal pathway when supported by the available evidence. We have added this definition in the Cause of Death Determination part of Methods section.

Among cases where intrauterine hypoxia was identified as the main fetal condition, 18% had no identifiable maternal or placental condition. In some of these cases, despite the absence of documented maternal or placental abnormalities, histopathological evidence supported the diagnosis of intrauterine hypoxia such as intrauterine fetal distress in lung tissue, and it was therefore assigned as the main fetal condition by the panel. We have added this interpretation in the result section where we mentioned about this initially.

- Consider presenting hypoxia stratified by timing (antepartum vs intrapartum) where timing is known, since prevention differs substantially.

**Response:** We have presented hypoxia stratified by timing (antepartum, intrapartum, and unknown) in Supplementary Table 9, including associated fetal and maternal conditions, fetal heartbeat status, and maceration. These findings have also been summarized in the Results section of the main manuscript.

- There is notable missingness: gestational age unknown for 21%, timing unknown for 24%, placenta unavailable for 51%, and fetal heartbeat documentation missing in >50% of cases.

1. Add a dedicated missingness table (overall and by site) for the key variables that drive classification and prevention recommendations (GA, timing, fetal heart monitoring, placenta availability, completeness of maternal records)

**Response:** We have reported the extent of missing or unknown data for key variables within Table 1. In addition, we have added Supplementary Table 10 to include a category showing cases in which maternal records were available but no identified maternal condition, to further clarify data completeness. Given that missingness is already presented within the main and supplementary tables, we did not create a separate missingness table. However, we would be happy to include one if the reviewer feels it would improve clarity.

2. Explicitly describe whether and how missingness influenced DeCoDe confidence levels (you note level-1 confidence for fetal condition in 81%; show how this varies by site and by major cause group).

**Response**: Based on this comment from the reviewer, we went back to examine relationships between the diagnoses and the confidence assigned to causes across sites. We noted some discrepancies between how sites were using the confident codes so have decided to exclude these from the paper. In this revision we have now provided more details about how causes were determined, which we hope will be sufficient for the reader. We also include in the discussion comments about missingness and how sites handled diagnoses with missing data.

3. Consider sensitivity analyses restricted to: 1) cases with placenta examined; and/or, 2) cases with known timing; and/or, 3) cases with GA known (for SGA-related analyses).

**Response:**

(1) We conducted sensitivity analyses restricted to cases with placental examination (Supplementary Table 5). While some differences were observed, the overall distribution of fetal and maternal conditions remained broadly consistent with the main analysis.

(2) We also conducted analyses restricted to cases with known timing of stillbirth (Supplementary Table 9), and the findings were consistent with the primary analysis, with expected differences between antepartum and intrapartum deaths.

(3) In addition, we performed sensitivity analyses stratified by gestational age to assess differences across pregnancy stages (Supplementary table 12). Intrauterine hypoxia remained the predominant fetal condition across all groups, while maternal conditions varied by gestational age, with hypertension and placental complications more common in preterm stillbirths and intrapartum-related conditions more frequent in term stillbirths.

These issues have now been addressed in the Discussion section.

- You report 72% of stillbirths are considered preventable, and that 85% of preventable deaths could be prevented via improved antenatal/obstetric care. This is policy-relevant but vulnerable to subjectivity and site-to-site interpretation differences. Define “preventable” explicitly: preventable under “optimal provision of existing care”—what constitutes “existing” across sites with different resources?

**Response:** We agree that clarification of the definition of “preventable” is essential, particularly given variation in health system capacity across sites. In CHAMPS, preventability is assessed within the local context and refers to deaths that could potentially be avoided through optimal delivery and use of the care available in that setting at the time of death.

We have now explicitly defined this in the Methods section (Cause of Death Determination section, second paragraph ) and further clarified in the Discussion that “existing care” varies across sites, which may influence interpretation and limit direct comparability. At the same time, this approach is aligned with the CHAMPS objective which is to provide evidence that can guide action in each local setting. For that reason, preventability should be interpreted within the context of each site.

- Provide information on standardization and inter-panel consistency: 1) training, decision rules, and whether cross-site calibration occurred; and 2) whether a subset of cases was double-reviewed across sites or centrally adjudicated.

**Response:** We have added additional details on standardization and inter-panel consistency in the methods section (Third paragraph, under cause of death determination section).

- Present preventability by cause group (hypoxia, congenital infection, congenital anomalies, etc.) and by timing (antepartum/intrapartum), because prevention levers differ. The subcategory expansion (more granular prevention categories) is good please add the actual category definitions in a supplement and summarize the top 5 subcategories in the main text for readability.

**Response:**

Preventability by cause group is presented in Table 2. In addition, we have expanded Supplementary Table 9 to include preventability stratified by timing of stillbirth (antepartum, intrapartum, and unknown), as recommended.

We have added the definitions of prevention subcategories to the Supplementary Table 1. In addition, we have summarized the most frequently identified prevention subcategories in the result section for prevention.

- Methods - In Methods, there is a sentence about exploring the relationship between foot length and gestational age and distributions for SGA estimation. This reads like an editing artifact and is confusing in a stillbirth MITS/DeCoDe paper. Remove this sentence unless it is central to your GA/SGA classification approach. If you keep it, clarify: Why was foot length used here? Was GA sometimes derived from anthropometry? If yes, specify the algorithm and validation, and how it impacts SGA classification.

**Response:**

We agree with the reviewer and have removed the sentence from the main text and also the supplementary figure.

- Methods - You identify multiple pathogens using TAC PCR and cultures, and list major pathogens (GBS, E. coli, Enterococcus). Since MITS-based pathogen detection can be sensitive, readers will want reassurance around contamination, colonization vs invasive infection, and causal inference. State the causal criteria for assigning congenital infection as cause (e.g., pathogen in sterile site + histopathology consistent with infection + clinical context). Describe contamination control steps for sampling and lab workflows, and how you handled polymicrobial detections (not uncommon in your data). Consider providing a breakdown of congenital infections by sterile-site vs non-sterile-site detection and by confidence level.

**Response:** We have expanded the Methods section to provide additional detail on contamination control, pathogen detection, and causal attribution of congenital infections (Second paragraph of method section).

*Consider providing a breakdown of congenital infections by sterile-site vs non-sterile-site detection and by confidence level-*

**Response:** All detected infections came from samples from sterile sites (CSF, blood, and lung tissue).

*Consider providing breakdown of congenital infection by confidence level*

**Response:**

As we have mentioned earlier in the response, we re-examined congenital infection by confidence level across sites. This review identified discrepancies in how confidence levels were applied, so we decided not to include this breakdown in the revised manuscript.

- The Findings are clear and compelling; consider adding a one-line note on consent rate and missingness to prevent overinterpretation of representativeness.

**Response:** A note on the consent rate and implications is in the first paragraph of the result section.

- Stillbirth definition is provided (≥1000g or ≥28 weeks) and death notification window described; please also specify whether multiple births were included and how they were handled.

**Response:** Multiple births were included and analyzed as independent MITS cases. As shown in Table 1, 8% of cases were from multiple gestations.

- Statistical analysis is described briefly; add which comparisons were planned a priori and consider adjusting for multiple comparisons if many site-wise tests are presented.

**Response:** Our objective was to describe the cases and compare findings across sites. These analyses were planned *a priori*.

- Ethics statement is concise; consider adding a sentence acknowledging cultural sensitivity around MITS and how community engagement supported consent processes (you cite that methods exist elsewhere).

**Response:** A statement acknowledging the cultural sensitivity of MITS and the role of community engagement in supporting consent processes has been added to the ethics section, consistent with previously described CHAMPS methods.

- Result - Table 1 is heavily relied upon; ensure it includes denominators for each key variable (e.g., fetal heartbeat documentation) and site-stratified missingness.

**Response:** We have added a footnote under Table 1 to clarify the denominator for each variable.

Result - For the statement that 95% delivered in a healthcare facility, discuss how CHAMPS case ascertainment might skew toward facility stillbirths and how community surveillance complements this.

**Response:** This issue is now addressed in the Discussion section (paragraph 7), where we clarify that the high proportion of facility-based deliveries likely reflects, in part, the CHAMPS surveillance and enrollment processes and compare this to the number of deliveries overall that occur in facilities at each site.

- Result - You mention 32 maternal deaths in clinical records without cause information—either remove or briefly interpret (e.g., indicates gaps in maternal chart documentation) and consider whether maternal death should be integrated into the causal chain where possible.

**Response:**  We have removed this information from the Results section, as detailed data were not available to adequately interpret these maternal deaths or determine how they should be integrated into the causal chain.

- Discussions - The discussion appropriately emphasizes geographic heterogeneity and care gaps. Consider strengthening the “what next” section with 2–3 priority, context-specific interventions mapped to the largest preventable fractions by region/site-type (even if kept high level). When stating that congenital infections may be higher in South Africa because care quality is better (thus fewer deaths from care gaps), frame this explicitly as a hypothesis and note alternative explanations (e.g., differential specimen handling, diagnostic intensity, panel tendencies).

**Response:** We have addressed this issue and revised the Discussion by adding a brief “what next” section highlighting priority, context-specific interventions aligned with the most common and preventable causes.

- The CHAMPS data access statement is included; consider stating what elements are publicly accessible vs require request, and whether de-identified MITS/pathology results are shareable.

**Response:** Deidentified data can be requested at <https://champshealth.org/> including MITS and pathology results. This information is available under the Data sharing section.
