# Supplementary material for: Causes of stillbirth in sub-Saharan Africa and South Asia: Findings from Child Health and Mortality Prevention Surveillance, 2016–2023
Source: PLOS Glob Public Health. 2026 Oct 27;6(10):e0005235. doi: 10.1371/journal.pgph.0005235 (PMC13712675; doi:10.1371/journal.pgph.0005235)
Supplement: Supplementary file 3 [file pgph.0005235.s001.zip › S1 File/Stillbirth supplementary tables_15 May 2026_AR_clean.docx]

**Supplementary appendix**

Supplement to: Rahman A, Lee K, Arifeen SE, et al. Causes of stillbirth in sub-Saharan Africa and South Asia: Findings from the Child Health and Mortality Prevention Surveillance Network, 2016-2023.

**Supplementary Table 1: Definitions of prevention subcategories derived from DeCoDe recommendations**

| **Main category** | **Subcategory** | **Definition** |
| --- | --- | --- |
| Improved antenatal care | Any improved antenatal care | General recommendation to improve antenatal care without specifying a particular component |
|  | Quality antenatal care | Provision of comprehensive, guideline-based antenatal services, including timely visits, risk identification, and appropriate clinical management |
|  | Management of medical conditions | Detection, monitoring, and treatment of maternal medical conditions during pregnancy, including hypertension, diabetes, and other comorbidities |
|  | Laboratory investigations | Appropriate use of laboratory tests during pregnancy to identify maternal or fetal conditions |
|  | Fetal screening and growth monitoring | Monitoring fetal growth and well-being during pregnancy, including assessment for fetal distress or growth restriction |
|  | Ultrasound by skilled sonographer | Use of ultrasound by trained personnel to assess fetal development, gestational age, and structural abnormalities |
|  | Health education | Counseling and education of pregnant women on danger signs, care-seeking, and healthy pregnancy practices |
| Improved obstetric care and management | Any improved obstetric care and management | General recommendation to improve intrapartum or delivery care without specifying a component |
|  | Delivery at health facility | Ensuring delivery occurs in a healthcare facility with skilled birth attendants |
|  | Access to timely emergency obstetric care | Availability and timely access to emergency obstetric interventions, including cesarean delivery and management of complications |
|  | Obstetric management | Appropriate clinical management during labor and delivery, including monitoring and timely intervention |
|  | Improved referral system | Strengthening referral pathways to ensure timely transfer of patients requiring higher-level care |
|  | Improved documentation of medical record | Accurate and complete recording of maternal and fetal clinical information during pregnancy and delivery |

**Supplementary Table 2. Number of stillbirths eligible for enrollment in CHAMPS, underwent minimal invasive tissue sampling, completed cause of death determination, and ascertained cause of death, by CHAMPS site, 2016-2023.**

|  | **All eligible for enrollment**  **N=4876**  **n (%)** | **Bangladesh**  **N=1356**  **n (%)** | **Ethiopia**  **N=935**  **n (%)** | **Kenya**  **N=394**  **n (%)** | **Mali**  **N=635**  **n (%)** | **Mozambique**  **N=719**  **n (%)** | **Sierra Leone**  **N=347**  **n (%)** | **South Africa**  **N=490**  **n (%)** |
| --- | --- | --- | --- | --- | --- | --- | --- | --- |
| Underwent minimally invasive tissue sampling | 2731 (56) | 432 (32) | 608 (65) | 323 (82) | 274 (43) | 507 (71) | 279 (80) | 308 (63) |
| Completed cause of death determination | 2492 (51) | 405 (30) | 568 (61) | 265 (67) | 212 (33) | 498 (69) | 238 (69) | 306 (62) |
| Ascertained cause of death | 2381 (49) | 396 (29) | 537 (57) | 262 (66) | 202 (32) | 470 (65) | 222 (64) | 292 (60) |

**Supplementary Figure 1. Number of stillbirths eligible for minimally invasive tissue sampling (N=4876), by year, site, and consent status.**

**Supplementary Table 3. Small for gestational age among 1726 stillbirths for whom we have data on gestational age, by CHAMPS site, 2016-2023**

| **Characteristics** | **Total**  **N=1726**  **n (%)** | **Bangladesh**  **N=354**  **n (%)** | **Ethiopia**  **N=178**  **n (%)** | **Kenya**  **N=237**  **n (%)** | **Mali**  **N=141**  **n (%)** | **Mozambique**  **N=363**  **n (%)** | **Sierra Leone**  **N=212**  **n (%)** | **South Africa**  **N=241**  **n (%)** |
| --- | --- | --- | --- | --- | --- | --- | --- | --- |
| *Small-for-gestational age |  |  |  |  |  |  |  |  |
| Yes | 625 (36) | 184 (52) | 61 (34) | 50 (21) | 56 (39) | 121 (33) | 57 (27) | 96 (40) |
| No | 1101 (64) | 170 (48) | 117 (66) | 187 (79) | 85 (60) | 242 (67) | 155 (73) | 145 (60) |

*Gestational age was not usually assessed by an early ultrasound

**Supplementary Table 4. Causes of death among 2492 stillbirth cases with complete cause of death determination, by CHAMPS site, 2016-2023**

| **Cause of death** | **All**  **N=2492**  **n (%)** | **Bangladesh**  **N=405**  **n (%)** | **Ethiopia**  **N=568**  **n (%)** | **Kenya**  **N=265**  **n (%)** | **Mali**  **N=212**  **n (%)** | **Mozambique**  **N=498**  **n (%)** | **Sierra Leone**  **N=238**  **n (%)** | **South Africa**  **N=306**  **n (%)** |
| --- | --- | --- | --- | --- | --- | --- | --- | --- |
| Main condition in the fetus |  |  |  |  |  |  |  |  |
| Intrauterine hypoxia | 1864 (75) | 347 (86) | 346 (61) | 236 (89) | 151 (71) | 395 (79) | 207 (87) | 182 (59) |
| Congenital birth defects | 218 (9) | 21 (5) | 134 (24) | 4 (2) | 11 (5) | 28 (6) | 7 (3) | 13 (4) |
| Congenital infection | 200 (8) | 24 (6) | 29 (5) | 10 (4) | 8 (4) | 39 (8) | 3 (1) | 87 (28) |
| Others | 60 (2) | 4 (1) | 24 (4) | 2 (0) | 19 (9) | 7 (1) | 0 (0) | 4 (2) |
| Undetermined | 150 (6) | 9 (2) | 35 (6) | 13 (5) | 23 (11) | 29 (6) | 21 (9) | 20 (7) |
| Other condition in the fetus^1^ |  |  |  |  |  |  |  |  |
| Intrauterine hypoxia | 113 (5) | 8 (2) | 74 (13) | 0 (0) | 20 (9) | 3 (1) | 5 (2) | 3 (1) |
| Intrauterine growth restriction | 53 (2) | 51 (13) | 0 (0) | 1 (0) | 0 (0) | 0 (0) | 0 (0) | 1 (0) |
| Congenital infection | 28 (1) | 2 (0) | 14 (2) | 0 (0) | 1 (0) | 0 (0) | 2 (1) | 9 (3) |
| Preterm birth complications | 25 (1) | 11 (3) | 2 (0) | 0 (0) | 0 (0) | 2 (0) | 10 (4) | 0 (0) |
| Congenital birth defects | 9 (0) | 6 (1) | 2 (0) | 0 (0) | 0 (0) | 1 (0) | 0 (0) | 0 (0) |
| Other | 6 (0) | 2 (0) | 1 (0) | 0 (0) | 0 (0) | 0 (0) | 2 (1) | 1 (0) |
| Aspiration syndrome | 2 (0) | 2 (0) | 0 (0) | 0 (0) | 0 (0) | 0 (0) | 0 (0) | 0 (0) |
| Birth trauma | 1 (0) | 0 (0) | 0 (0) | 0 (0) | 0 (0) | 0 (0) | 1 (0) | 0 (0) |
| Main maternal condition (ICD-PM) |  |  |  |  |  |  |  |  |
| M1: Complications of placenta, cord and membranes | 724 (29) | 91 (22) | 102 (18) | 83 (31) | 92 (43) | 157 (32) | 79 (33) | 120 (39) |
| M2: Complications of pregnancy | 131 (5) | 32 (8) | 30 (5) | 15 (6) | 24 (11) | 12 (2) | 10 (4) | 8 (3) |
| M3: Other complications of labor and delivery | 319 (13) | 42 (10) | 47 (8) | 47 (18) | 21 (10) | 109 (22) | 44 (18) | 9 (3) |
| M4: Medical and surgical condition | 602 (24) | 158 (39) | 86 (15) | 70 (26) | 39 (18) | 79 (16) | 49 (21) | 121 (40) |
| Other maternal condition | 18 (1) | 4 (1) | 1 (0) | 1 (0) | 4 (2) | 0 (0) | 0 (0) | 8 (3) |
| No condition identified | 698 (28) | 78 (19) | 302 (53) | 49 (18) | 32 (15) | 141 (28) | 56 (24) | 40 (13) |
| Etiologic agents |  |  |  |  |  |  |  |  |
| Gram-positive bacteria | 102 (4) | 8 (2) | 18 (3) | 4 (2) | 5 (2) | 8 (2) | 1 (0) | 58 (19) |
| Gram-negative bacteria | 116 (5) | 11 (3) | 21 (4) | 3 (1) | 8 (4) | 8 (2) | 3 (1) | 62 (20) |
| Gram-variable bacteria | 1 (0) | 0 (0) | 0 (0) | 0 (0) | 0 (0) | 0 (0) | 0 (0) | 1 (0) |
| Viruses | 23 (1) | 6 (1) | 3 (1) | 1 (0) | 0 (0) | 7 (1) | 0 (0) | 6 (2) |
| Fungi | 1 (0) | 0 (0) | 0 (0) | 0 (0) | 0 (0) | 0 (0) | 1 (0) | 0 (0) |
| Protozoa | 2 (0) | 0 (0) | 2 (0) | 0 (0) | 0 (0) | 0 (0) | 0 (0) | 0 (0) |

^1^2278 stillbirths had no other condition in the fetus and 23 stillbirths had two other conditions in the fetus.

**Supplementary Table 5. Main condition in the fetus attributed to death among 2492 stillbirths with complete cause of death determination, by whether placenta specimen was collected, 2016-2023.**

| **Characteristic** | **Total**  **N=2492**  **n (%)** | **Placenta collected**  **N=1224**  **n (%)** | **Placenta not collected**  **N=1268**  **n (%)** |
| --- | --- | --- | --- |
| **Main condition in the fetus** |  |  |  |
| Intrauterine hypoxia | 1864 (75) | 955 (78) | 909 (72) |
| Congenital birth defects | 218 (9) | 79 (6) | 139 (11) |
| Congenital infection | 200 (8) | 106 (9) | 94 (7) |
| Intra-uterine growth restriction | 32 (1) | 17 (1) | 15 (1) |
| Preterm birth complications | 6 (0) | 2 (0) | 4 (0) |
| Aspiration syndrome | 2 (0) | 0 (0) | 2 (0) |
| Birth trauma | 1 (0) | 1 (0) | 0 (0) |
| Cancer | 1 (0) | 0 (0) | 1 (0) |
| Poisoning | 1 (0) | 0 (0) | 1 (0) |
| Other | 17 (1) | 10 (1) | 7 (1) |
| Undetermined | 150 (6) | 54 (4) | 96 (8) |
| **Main maternal condition** |  |  |  |
| Placental complications | 446 (18) | 273 (22) | 173 (14) |
| Maternal hypertension | 414 (17) | 205 (17) | 209 (16) |
| Chorioamnionitis and membrane complications | 173 (7) | 136 (11) | 37 (3) |
| Obstructed labor and fetal malpresentation | 143 (6) | 43 (4) | 100 (8) |
| Other factor (M3) | 133 (5) | 64 (5) | 69 (5) |
| Umbilical cord complications | 105 (4) | 49 (4) | 56 (4) |
| Other factor (M4) | 64 (3) | 42 (3) | 22 (2) |
| Multiple gestation | 59 (2) | 19 (2) | 40 (3) |
| Maternal infection | 58 (2) | 31 (3) | 27 (2) |
| Premature rupture of membranes | 34 (1) | 12 (1) | 22 (2) |
| Maternal diabetes | 31 (1) | 20 (2) | 11 (1) |
| Uterine fluid disorders | 23 (1) | 15 (1) | 8 (1) |
| Prolonged pregnancy | 18 (1) | 9 (1) | 9 (1) |
| Uterine rupture | 18 (1) | 9 (1) | 9 (1) |
| Other factor (Other) | 15 (1) | 6 (0) | 9 (1) |
| Other factor (M2) | 11 (0) | 4 (0) | 7 (1) |
| HIV | 9 (0) | 5 (0) | 4 (0) |
| Maternal injury and accident | 7 (0) | 4 (0) | 3 (0) |
| Preterm labor or delivery | 6 (0) | 2 (0) | 4 (0) |
| Cervical insufficiency and pelvic anomalies | 4 (0) | 1 (0) | 3 (0) |
| Maternal nutritional disorders | 4 (0) | 3 (0) | 1 (0) |
| Maternal circulatory and respiratory diseases | 3 (0) | 2 (0) | 1 (0) |
| Sickle cell disorders | 3 (0) | 2 (0) | 1 (0) |
| Anemias | 2 (0) | 0 (0) | 2 (0) |
| Obesity | 2 (0) | 1 (0) | 1 (0) |
| Other endocrine, metabolic, blood, and immune disorders | 2 (0) | 2 (0) | 0 (0) |
| Poisoning | 2 (0) | 0 (0) | 2 (0) |
| Cesarean delivery | 1 (0) | 1 (0) | 0 (0) |
| Heart Diseases | 1 (0) | 1 (0) | 0 (0) |
| Liver disease | 1 (0) | 0 (0) | 1 (0) |
| Maternal medication or toxic exposure | 1 (0) | 1 (0) | 0 (0) |
| Other respiratory disease | 1 (0) | 1 (0) | 0 (0) |
| No condition identified | 698 (28) | 261 (21) | 437 (34) |
| **Stillbirth type** |  |  |  |
| Fresh | 1282 (51) | 586 (48) | 696 (55) |
| Macerated | 1120 (45) | 607 (50) | 513 (40) |
| Unknown | 90 (4) | 31 (3) | 59 (5) |
| **Stillbirth timing** |  |  |  |
| Antepartum | 1077 (43) | 602 (49) | 475 (37) |
| Intrapartum | 825 (33) | 353 (29) | 472 (37) |
| Unknown | 590 (24) | 269 (22) | 321 (25) |

**Supplementary Table 6. Main maternal conditions among stillbirths with intrauterine hypoxia, congenital infection, and congenital birth defects as main condition in the fetus.**

| **Main maternal condition** | | **All Stillbirths**  **N=2492** | **Intrauterine hypoxia**  **N=1864**  **n (%)** | **Congenital birth defects**  **N=218**  **n (%)** | **Congenital infections**  **N=200**  **n (%)** |
| --- | --- | --- | --- | --- | --- |
| **ICD-PM Group** | **Specific** |  |  |  |  |
| M1: Complications of placenta, cord and membranes | Placental complications | 446 (18) | 415 (22) | 1 (0) | 7 (4) |
|  | Chorioamnionitis and membrane complications | 173 (7) | 92 (5) | 2 (1) | 73 (36) |
|  | Umbilical cord complications | 105 (4) | 103 (6) | 0 (0) | 1 (0) |
| M2: Complications of pregnancy | Multiple gestation | 59 (2) | 44 (2) | 4 (2) | 0 (0) |
|  | Premature rupture of membranes | 34 (1) | 23 (1) | 0 (0) | 11 (6) |
|  | Uterine fluid disorders | 23 (1) | 20 (1) | 1 (0) | 2 (1) |
|  | Other factor (M2) | 11 (0) | 5 (0) | 0 (0) | 1 (0) |
|  | Cervical insufficiency and pelvic anomalies | 4 (0) | 2 (0) | 0 (0) | 2 (1) |
| M3: Other complications of labor and delivery | Obstructed labor and fetal malpresentation | 143 (6) | 133 (7) | 6 (3) | 2 (1) |
|  | Other factor (M3) | 133 (5) | 129 (7) | 1 (0) | 3 (2) |
|  | Prolonged pregnancy | 18 (1) | 17 (1) | 0 (0) | 0 (0) |
|  | Uterine rupture | 18 (1) | 17 (1) | 1 (0) | 0 (0) |
|  | Preterm labor or delivery | 6 (0) | 6 (0) | 0 (0) | 0 (0) |
|  | Cesarean delivery | 1 (0) | 1 (0) | 0 (0) | 0 (0) |
| M4: Medical and surgical condition | Maternal hypertension | 414 (17) | 382 (20) | 3 (1) | 13 (6) |
|  | Other factor (M4) | 64 (3) | 54 (3) | 0 (0) | 6 (3) |
|  | Maternal infection | 58 (2) | 30 (2) | 1 (0) | 25 (12) |
|  | Maternal diabetes | 31 (1) | 22 (1) | 3 (1) | 4 (2) |
|  | HIV | 9 (0) | 6 (0) | 0 (0) | 3 (2) |
|  | Maternal injury and accident | 7 (0) | 6 (0) | 0 (0) | 0 (0) |
|  | Maternal nutritional disorders | 4 (0) | 3 (0) | 1 (0) | 0 (0) |
|  | Maternal circulatory and respiratory diseases | 3 (0) | 3 (0) | 0 (0) | 0 (0) |
|  | Sickle cell disorders | 3 (0) | 2 (0) | 0 (0) | 0 (0) |
|  | Anemias | 2 (0) | 1 (0) | 0 (0) | 0 (0) |
|  | Obesity | 2 (0) | 2 (0) | 0 (0) | 0 (0) |
|  | Other endocrine, metabolic, blood, and immune disorders | 2 (0) | 2 (0) | 0 (0) | 0 (0) |
|  | Heart Diseases | 1 (0) | 1 (0) | 0 (0) | 0 (0) |
|  | Liver disease | 1 (0) | 1 (0) | 0 (0) | 0 (0) |
|  | Other respiratory disease | 1 (0) | 1 (0) | 0 (0) | 0 (0) |
| Other maternal conditions^1^ | Other maternal conditions^1^ | 18 (1) | 9 (0) | 3 (1) | 3 (2) |
| No maternal condition identified | No maternal condition identified | 698 (28) | 332 (18) | 191 (88) | 44 (22) |

^1^Includes M35.9, O04, P04.1, P96.4, QA43.Y, QA49, R45.6, T65.9, X68, Y66, Z35.3, Z35.5, Z35.6, Z55.9, Z87.5

**Supplementary Table 7: Etiologic agents among stillbirths, all CHAMPS sites, 2016-2023**

| **Etiologic agent** | **All**  **N=2492**  **n (%)** | **Bangladesh**  **N=405**  **n (%)** | **Ethiopia**  **N=568**  **n (%)** | **Kenya**  **N=265**  **n (%)** | **Mali**  **N=212**  **n (%)** | **Mozambique**  **N=498**  **n (%)** | **Sierra Leone**  **N=238**  **n (%)** | **South Africa**  **N=306**  **n (%)** |
| --- | --- | --- | --- | --- | --- | --- | --- | --- |
| Gram-positive bacteria | 102 (4) | 8 (2) | 18 (3) | 4 (2) | 5 (2) | 8 (2) | 1 (0) | 58 (19) |
| Group B Streptococcus | 46 (2) | 4 (1) | 9 (2) | 2 (1) | 1 (0) | 4 (1) | 0 (0) | 26 (8) |
| *Enterococcus faecalis* | 24 (1) | 1 (0) | 0 (0) | 0 (0) | 0 (0) | 0 (0) | 0 (0) | 23 (8) |
| *Streptococcus* spp. | 12 (0) | 0 (0) | 4 (1) | 2 (1) | 1 (0) | 3 (1) | 1 (0) | 1 (0) |
| *Streptococcus pneumoniae* | 4 (0) | 0 (0) | 0 (0) | 0 (0) | 2 (1) | 1 (0) | 0 (0) | 1 (0) |
| *Enterococcus* spp. | 3 (0) | 0 (0) | 1 (0) | 0 (0) | 1 (0) | 0 (0) | 0 (0) | 1 (0) |
| *Staphylococcus aureus* | 3 (0) | 0 (0) | 0 (0) | 0 (0) | 0 (0) | 0 (0) | 0 (0) | 3 (1) |
| Group A Streptococcus | 2 (0) | 0 (0) | 2 (0) | 0 (0) | 0 (0) | 0 (0) | 0 (0) | 0 (0) |
| *Bacillus* spp. | 1 (0) | 0 (0) | 0 (0) | 0 (0) | 0 (0) | 0 (0) | 0 (0) | 1 (0) |
| Coagulase negative Staphylococcus | 1 (0) | 0 (0) | 0 (0) | 0 (0) | 0 (0) | 0 (0) | 0 (0) | 1 (0) |
| *Enterococcus faecium* | 1 (0) | 0 (0) | 1 (0) | 0 (0) | 0 (0) | 0 (0) | 0 (0) | 0 (0) |
| Gram positive bacteria (unspecified) | 1 (0) | 0 (0) | 1 (0) | 0 (0) | 0 (0) | 0 (0) | 0 (0) | 0 (0) |
| *Listeria monocytogenes* | 1 (0) | 1 (0) | 0 (0) | 0 (0) | 0 (0) | 0 (0) | 0 (0) | 0 (0) |
| *Staphylococcus* spp. | 1 (0) | 1 (0) | 0 (0) | 0 (0) | 0 (0) | 0 (0) | 0 (0) | 0 (0) |
| *Streptococcus gallolyticus* ssp. *pasteurianus* | 1 (0) | 1 (0) | 0 (0) | 0 (0) | 0 (0) | 0 (0) | 0 (0) | 0 (0) |
| Viridans Streptococcus | 1 (0) | 0 (0) | 0 (0) | 0 (0) | 0 (0) | 0 (0) | 0 (0) | 1 (0) |
| Gram-negative bacteria | 116 (5) | 11 (3) | 21 (4) | 3 (1) | 8 (4) | 8 (2) | 3 (1) | 62 (20) |
| *Escherichia coli* | 43 (2) | 0 (0) | 9 (2) | 1 (0) | 3 (1) | 0 (0) | 0 (0) | 30 (10) |
| *Treponema pallidum* | 20 (1) | 2 (0) | 0 (0) | 0 (0) | 0 (0) | 4 (1) | 0 (0) | 14 (5) |
| *Klebsiella pneumoniae* | 15 (1) | 0 (0) | 5 (1) | 0 (0) | 1 (0) | 2 (0) | 1 (0) | 6 (2) |
| *Ureaplasma* spp. | 12 (0) | 4 (1) | 1 (0) | 1 (0) | 1 (0) | 0 (0) | 1 (0) | 4 (1) |
| *Escherichia coli*/*Shigella* spp. | 8 (0) | 0 (0) | 4 (1) | 0 (0) | 1 (0) | 1 (0) | 0 (0) | 2 (1) |
| *Haemophilus influenzae* | 5 (0) | 2 (0) | 1 (0) | 0 (0) | 2 (1) | 0 (0) | 0 (0) | 0 (0) |
| *Enterobacter cloacae* | 4 (0) | 0 (0) | 0 (0) | 0 (0) | 0 (0) | 0 (0) | 0 (0) | 4 (1) |
| *Acinetobacter baumannii* | 1 (0) | 0 (0) | 0 (0) | 0 (0) | 0 (0) | 0 (0) | 1 (0) | 0 (0) |
| *Cronubacter sakazakii* | 1 (0) | 1 (0) | 0 (0) | 0 (0) | 0 (0) | 0 (0) | 0 (0) | 0 (0) |
| *Escherichia fergusonii* | 1 (0) | 0 (0) | 1 (0) | 0 (0) | 0 (0) | 0 (0) | 0 (0) | 0 (0) |
| *Klebsiella oxytoca* | 1 (0) | 0 (0) | 0 (0) | 0 (0) | 0 (0) | 0 (0) | 0 (0) | 1 (0) |
| *Neisseria gonorrhoeae* | 1 (0) | 1 (0) | 0 (0) | 0 (0) | 0 (0) | 0 (0) | 0 (0) | 0 (0) |
| *Prevotella bivia* | 1 (0) | 0 (0) | 0 (0) | 0 (0) | 0 (0) | 0 (0) | 0 (0) | 1 (0) |
| *Pseudomonas aeruginosa* | 1 (0) | 1 (0) | 0 (0) | 0 (0) | 0 (0) | 0 (0) | 0 (0) | 0 (0) |
| *Sneathia amnii* | 1 (0) | 0 (0) | 0 (0) | 0 (0) | 0 (0) | 1 (0) | 0 (0) | 0 (0) |
| *Ureaplasma urealyticum* | 1 (0) | 0 (0) | 0 (0) | 1 (0) | 0 (0) | 0 (0) | 0 (0) | 0 (0) |
| Gram-variable bacteria | 1 (0) | 0 (0) | 0 (0) | 0 (0) | 0 (0) | 0 (0) | 0 (0) | 1 (0) |
| *Gardnerella vaginalis* | 1 (0) | 0 (0) | 0 (0) | 0 (0) | 0 (0) | 0 (0) | 0 (0) | 1 (0) |
| Viruses | 23 (1) | 6 (1) | 3 (1) | 1 (0) | 0 (0) | 7 (1) | 0 (0) | 6 (2) |
| Cytomegalovirus | 20 (1) | 4 (1) | 2 (0) | 1 (0) | 0 (0) | 7 (1) | 0 (0) | 6 (2) |
| Adenovirus | 1 (0) | 1 (0) | 0 (0) | 0 (0) | 0 (0) | 0 (0) | 0 (0) | 0 (0) |
| Parvovirus B19 | 1 (0) | 0 (0) | 1 (0) | 0 (0) | 0 (0) | 0 (0) | 0 (0) | 0 (0) |
| Varicella zoster virus | 1 (0) | 1 (0) | 0 (0) | 0 (0) | 0 (0) | 0 (0) | 0 (0) | 0 (0) |
| Fungi | 1 (0) | 0 (0) | 0 (0) | 0 (0) | 0 (0) | 0 (0) | 1 (0) | 0 (0) |
| *Candida albicans* | 1 (0) | 0 (0) | 0 (0) | 0 (0) | 0 (0) | 0 (0) | 1 (1) | 0 (0) |
| Protozoa | 2 (0) | 0 (0) | 2 (0) | 0 (0) | 0 (0) | 0 (0) | 0 (0) | 0 (0) |
| *Toxoplasma gondii* | 2 (0) | 0 (0) | 2 (0) | 0 (0) | 0 (0) | 0 (0) | 0 (0) | 0 (0) |

**Supplementary Table 8: Type and timing of stillbirth by CHAMPS sites, 2016-2023**

|  | **Bangladesh**  **N=405**  **n (%)** | | **Ethiopia**  **N=568**  **n (%)** | | **Kenya**  **N=265**  **n (%)** | | **Mali**  **N=212**  **n (%)** | | **Mozambique**  **N=498**  **n (%)** | | **Sierra Leone**  **N=238**  **n (%)** | | **South Africa**  **N=306**  **n (%)** | |
| --- | --- | --- | --- | --- | --- | --- | --- | --- | --- | --- | --- | --- | --- | --- |
|  | Maceration | Fresh | Maceration | Fresh | Maceration | Fresh | Maceration | Fresh | Maceration | Fresh | Maceration | Fresh | Maceration | Fresh |
| Antepartum | 185 (45) | 41 (10) | 95 (17) | 75 (13) | 49 (18) | 24 (9) | 60 (28) | 29 (13) | 95 (19) | 87 (17) | 78 (33) | 26 (11) | 162 (53) | 40 (13) |
| Intrapartum | 29 (7) | 100 (25) | 70 (12) | 156 (27) | 38 (14) | 52 (19) | 4 (2) | 37 (17) | 19 (4) | 94 (19) | 15 (6) | 108 (45) | 23 (7) | 38 (12) |

**Supplementary Table 9: Main condition in the fetus, main maternal condition, and preventability by stillbirth timing, 2016-2023**

|  | **Total**  **N=2492**  **n (%)** | **Antepartum**  **N=1077**  **n (%)** | **Intrapartum**  **N=825**  **n (%)** | **Unknown**  **N=590**  **n (%)** |
| --- | --- | --- | --- | --- |
| Main condition in the fetus |  |  |  |  |
| Intrauterine ant | 1864 (75) | 801 (74) | 650 (79) | 413 (70) |
| Congenital birth defects | 218 (9) | 69 (6) | 86 (10) | 63 (11) |
| Congenital infection | 200 (8) | 102 (9) | 48 (6) | 50 (8) |
| Intrauterine growth restriction | 32 (1) | 15 (1) | 8 (1) | 9 (2) |
| Preterm birth complications | 6 (0) | 3 (0) | 1 (0) | 2 (0) |
| Aspiration syndrome | 2 (0) | 0 (0) | 0 (0) | 2 (0) |
| Birth trauma | 1 (0) | 0 (0) | 1 (0) | 0 (0) |
| Cancer | 1 (0) | 1 (0) | 0 (0) | 0 (0) |
| Poisoning | 1 (0) | 1 (0) | 0 (0) | 0 (0) |
| Other | 17 (1) | 6 (1) | 7 (1) | 4 (1) |
| Undetermined | 150 (6) | 79 (7) | 24 (3) | 47 (8) |
| Main maternal condition (ICD-PM group) |  |  |  |  |
| M1: Complications of placenta, cord and membranes | 724 (29) | 345 (32) | 216 (26) | 163 (28) |
| M2: Complications of pregnancy | 131 (5) | 59 (5) | 44 (5) | 28 (5) |
| M3: Other complications of labor and delivery | 319 (13) | 43 (4) | 213 (26) | 63 (11) |
| M4: Medical and surgical condition | 602 (24) | 331 (31) | 156 (19) | 115 (19) |
| Other maternal condition | 18 (1) | 13 (1) | 4 (0) | 1 (0) |
| No maternal condition identified | 698 (28) | 286 (27) | 192 (23) | 220 (37) |
| Fetal heartbeat during labor |  |  |  |  |
| Present | 342 (14) | 57 (5) | 202 (24) | 83 (14) |
| Absent | 783 (31) | 398 (37) | 249 (30) | 136 (23) |
| Data missing | 1367 (55) | 622 (58) | 374 (45) | 371 (63) |
| Stillbirth type |  |  |  |  |
| Fresh | 1282 (51) | 322 (30) | 585 (71) | 375 (64) |
| Macerated | 1120 (45) | 724 (67) | 198 (24) | 198 (33) |
| Unknown | 90 (4) | 31 (3) | 42 (5) | 17 (3) |
| Preventability |  |  |  |  |
| Preventable given current situation | 1531 (61) | 588 (55) | 590 (72) | 353 (60) |
| Preventable given ideal situation | 277 (11) | 131 (12) | 79 (10) | 67 (11) |
| Not preventable | 499 (20) | 277 (26) | 104 (13) | 118 (20) |
| Not possible to determine with current data | 185 (7) | 81 (8) | 52 (6) | 52 (9) |

**Supplementary Table 10: Maternal medical record availability and maternal condition identification among 2492 stillbirths, 2016-2023.**

| **Category** | **All**  **N=2492**  **n (%)** | **Bangladesh**  **N=405**  **n (%)** | **Ethiopia**  **N=568**  **n (%)** | **Kenya**  **N=265**  **n (%)** | **Mali**  **N=212**  **n (%)** | **Mozambique**  **N=498**  **n (%)** | **Sierra Leone**  **N=238**  **n (%)** | **South Africa**  **N=306**  **n (%)** |
| --- | --- | --- | --- | --- | --- | --- | --- | --- |
| Has medical record and  maternal condition identified | 1639 (66) | 320 (79) | 233 (41) | 215 (81) | 147 (69) | 313 (63) | 178 (75) | 233 (76) |
| Has medical record and  no maternal condition identified | 576 (23) | 78 (19) | 223 (39) | 49 (18) | 24 (11) | 112 (22) | 55 (23) | 35 (11) |
| No medical record and  maternal condition identified | 155 (6) | 7 (2) | 33 (6) | 1 (0) | 33 (16) | 44 (9) | 4 (2) | 33 (11) |
| No medical record and  no maternal condition identified | 122 (5) | 0 (0) | 79 (14) | 0 (0) | 8 (4) | 29 (6) | 1 (0) | 5 (2) |

**Supplementary Figure 2 : Proportion of stillbirths with a main maternal condition identified and proportion with a placenta collected by sites, 2016-2023 (N=2492)**

**Supplementary Table 11. Main maternal conditions among stillbirths by small-for-gestational age.**

| **Main maternal condition** | | **All Stillbirths**  **N=2492** | **Small-for-gestational age**  **N=625**  **n (%)** | **Not small-for-gestational age**  **N=1101**  **n (%)** | **Unknown**  **N=766**  **n (%)** |
| --- | --- | --- | --- | --- | --- |
| **ICD-PM Group** | **Specific** |  |  |  |  |
| M1: Complications of placenta, cord and membranes | Placental complications | 446 (18) | 129 (21) | 209 (19) | 108 (14) |
|  | Chorioamnionitis and membrane complications | 173 (7) | 45 (7) | 89 (8) | 39 (5) |
|  | Umbilical cord complications | 105 (4) | 19 (3) | 53 (5) | 33 (4) |
| M2: Complications of pregnancy | Multiple gestation | 59 (2) | 18 (3) | 14 (1) | 27 (4) |
|  | Premature rupture of membranes | 34 (1) | 3 (0) | 18 (2) | 13 (2) |
|  | Uterine fluid disorders | 23 (1) | 12 (2) | 7 (1) | 4 (1) |
|  | Other factor (M2) | 11 (0) | 0 (0) | 7 (1) | 4 (1) |
|  | Cervical insufficiency and pelvic anomalies | 4 (0) | 1 (0) | 2 (0) | 1 (0) |
| M3: Other complications of labor and delivery | Obstructed labor and fetal malpresentation | 143 (6) | 20 (3) | 75 (7) | 48 (6) |
|  | Other factor (M3) | 133 (5) | 18 (3) | 76 (7) | 39 (5) |
|  | Prolonged pregnancy | 18 (1) | 7 (1) | 5 (0) | 6 (1) |
|  | Uterine rupture | 18 (1) | 0 (0) | 14 (1) | 4 (1) |
|  | Preterm labor or delivery | 6 (0) | 1 (0) | 3 (0) | 2 (0) |
|  | Cesarean delivery | 1 (0) | 1 (0) | 0 (0) | 0 (0) |
| M4: Medical and surgical condition | Maternal hypertension | 414 (17) | 141 (23) | 185 (17) | 88 (11) |
|  | Other factor (M4) | 64 (3) | 15 (2) | 40 (4) | 9 (1) |
|  | Maternal infection | 58 (2) | 18 (3) | 31 (3) | 9 (1) |
|  | Maternal diabetes | 31 (1) | 6 (1) | 17 (2) | 8 (1) |
|  | HIV | 9 (0) | 1 (0) | 7 (1) | 1 (0) |
|  | Maternal injury and accident | 7 (0) | 3 (0) | 3 (0) | 1 (0) |
|  | Maternal nutritional disorders | 4 (0) | 3 (0) | 0 (0) | 1 (0) |
|  | Maternal circulatory and respiratory diseases | 3 (0) | 2 (0) | 1 (0) | 0 (0) |
|  | Sickle cell disorders | 3 (0) | 1 (0) | 2 (0) | 0 (0) |
|  | Anemias | 2 (0) | 0 (0) | 2 (0) | 0 (0) |
|  | Obesity | 2 (0) | 0 (0) | 2 (0) | 0 (0) |
|  | Other endocrine, metabolic, blood, and immune disorders | 2 (0) | 1 (0) | 1 (0) | 0 (0) |
|  | Heart Diseases | 1 (0) | 0 (0) | 0 (0) | 1 (0) |
|  | Liver disease | 1 (0) | 0 (0) | 0 (0) | 1 (0) |
|  | Other respiratory disease | 1 (0) | 0 (0) | 0 (0) | 1 (0) |
| Other maternal conditions^1^ | Other maternal conditions^1^ | 18 (1) | 5 (1) | 8 (1) | 5 (1) |
| No maternal condition identified | No maternal condition identified | 698 (28) | 155 (25) | 230 (21) | 313 (41) |

^1^Includes M35.9, O04, P04.1, P96.4, QA43.Y, QA49, R45.6, T65.9, X68, Y66, Z35.3, Z35.5, Z35.6, Z55.9, Z87.5

**Supplementary Table 12: Main condition in the fetus and main maternal condition by gestational age**

| **Characteristic** | **Total**  **N=2492**  **n (%)** | **Preterm**  **N=1136**  **n (%)** | **Term**  **N=777**  **n (%)** | **Postdate**  **N=53**  **n (%)** | **Unknown**  **N=526**  **n (%)** |
| --- | --- | --- | --- | --- | --- |
| **Main condition in the fetus** |  |  |  |  |  |
| Intrauterine hypoxia | 1864 (75) | 854 (75) | 633 (81) | 43 (81) | 334 (63) |
| Congenital birth defects | 218 (9) | 92 (8) | 42 (5) | 5 (9) | 79 (15) |
| Congenital infection | 200 (8) | 105 (9) | 50 (6) | 2 (4) | 43 (8) |
| Intra-uterine growth restriction | 32 (1) | 8 (1) | 17 (2) | 0 (0) | 7 (1) |
| Preterm birth complications | 6 (0) | 4 (0) | 1 (0) | 0 (0) | 1 (0) |
| Aspiration syndrome | 2 (0) | 2 (0) | 0 (0) | 0 (0) | 0 (0) |
| Birth trauma | 1 (0) | 0 (0) | 1 (0) | 0 (0) | 0 (0) |
| Cancer | 1 (0) | 0 (0) | 1 (0) | 0 (0) | 0 (0) |
| Poisoning | 1 (0) | 1 (0) | 0 (0) | 0 (0) | 0 (0) |
| Other | 17 (1) | 11 (1) | 4 (1) | 0 (0) | 2 (0) |
| Undetermined | 150 (6) | 59 (5) | 28 (4) | 3 (6) | 60 (11) |
| **Main maternal condition** |  |  |  |  |  |
| Placental complications | 446 (18) | 246 (22) | 120 (15) | 4 (8) | 76 (14) |
| Maternal hypertension | 414 (17) | 255 (22) | 100 (13) | 4 (8) | 55 (10) |
| Chorioamnionitis and membrane complications | 173 (7) | 77 (7) | 69 (9) | 4 (8) | 23 (4) |
| Obstructed labor and fetal malpresentation | 143 (6) | 25 (2) | 85 (11) | 5 (9) | 28 (5) |
| Other factor (M3) | 133 (5) | 36 (3) | 63 (8) | 9 (17) | 25 (5) |
| Umbilical cord complications | 105 (4) | 28 (2) | 57 (7) | 3 (6) | 17 (3) |
| Other factor (M4) | 64 (3) | 46 (4) | 15 (2) | 0 (0) | 3 (1) |
| Multiple gestation | 59 (2) | 16 (1) | 23 (3) | 0 (0) | 20 (4) |
| Maternal infection | 58 (2) | 39 (3) | 12 (2) | 1 (2) | 6 (1) |
| Premature rupture of membranes | 34 (1) | 24 (2) | 0 (0) | 0 (0) | 10 (2) |
| Maternal diabetes | 31 (1) | 11 (1) | 14 (2) | 1 (2) | 5 (1) |
| Uterine fluid disorders | 23 (1) | 12 (1) | 6 (1) | 2 (4) | 3 (1) |
| Prolonged pregnancy | 18 (1) | 0 (0) | 8 (1) | 8 (15) | 2 (0) |
| Uterine rupture | 18 (1) | 4 (0) | 11 (1) | 1 (2) | 2 (0) |
| Other factor (Other) | 15 (1) | 9 (1) | 2 (0) | 0 (0) | 4 (1) |
| Other factor (M2) | 11 (0) | 6 (1) | 2 (0) | 1 (2) | 2 (0) |
| HIV | 9 (0) | 5 (0) | 3 (0) | 0 (0) | 1 (0) |
| Maternal injury and accident | 7 (0) | 5 (0) | 1 (0) | 0 (0) | 1 (0) |
| Preterm labor or delivery | 6 (0) | 5 (0) | 0 (0) | 0 (0) | 1 (0) |
| Cervical insufficiency and pelvic anomalies | 4 (0) | 4 (0) | 0 (0) | 0 (0) | 0 (0) |
| Maternal nutritional disorders | 4 (0) | 3 (0) | 0 (0) | 0 (0) | 1 (0) |
| Maternal circulatory and respiratory diseases | 3 (0) | 2 (0) | 1 (0) | 0 (0) | 0 (0) |
| Sickle cell disorders | 3 (0) | 3 (0) | 0 (0) | 0 (0) | 0 (0) |
| Anemias | 2 (0) | 1 (0) | 1 (0) | 0 (0) | 0 (0) |
| Obesity | 2 (0) | 0 (0) | 2 (0) | 0 (0) | 0 (0) |
| Other endocrine, metabolic, blood, and immune disorders | 2 (0) | 2 (0) | 0 (0) | 0 (0) | 0 (0) |
| Poisoning | 2 (0) | 1 (0) | 0 (0) | 0 (0) | 1 (0) |
| Cesarean delivery | 1 (0) | 0 (0) | 1 (0) | 0 (0) | 0 (0) |
| Heart Diseases | 1 (0) | 0 (0) | 0 (0) | 0 (0) | 1 (0) |
| Liver disease | 1 (0) | 0 (0) | 0 (0) | 0 (0) | 1 (0) |
| Maternal medication or toxic exposure | 1 (0) | 1 (0) | 0 (0) | 0 (0) | 0 (0) |
| Other respiratory disease | 1 (0) | 1 (0) | 0 (0) | 0 (0) | 0 (0) |
| No condition identified | 698 (28) | 269 (24) | 181 (23) | 10 (19) | 238 (45) |
|  |  |  |  |  |  |
| **Stillbirth type** |  |  |  |  |  |
| Fresh | 1282 (51) | 494 (43) | 461 (59) | 34 (64) | 293 (56) |
| Macerated | 1120 (45) | 605 (53) | 286 (37) | 18 (34) | 211 (40) |
| Unknown | 90 (4) | 37 (3) | 30 (4) | 1 (2) | 22 (4) |
| **Stillbirth timing** |  |  |  |  |  |
|  | 1077 (43) | 607 (53) | 295 (38) | 17 (32) | 158 (30) |
| Intrapartum | 825 (33) | 327 (29) | 320 (41) | 16 (30) | 162 (31) |
| Unknown | 590 (24) | 202 (18) | 162 (21) | 20 (38) | 206 (39) |

**Supplementary Table 13. Recommendations for how stillbirths could have been prevented among those that were deemed preventable, by CHAMPS site, 2016-2023 (N=1808)**

| **Recommendations^1^** | **Total**  **N=1808**  **n (%)** | **Bangladesh**  **N=364**  **n (%)** | **Ethiopia**  **N=470**  **n (%)** | **Kenya**  **N=250**  **n (%)** | **Mali**  **N=146**  **n (%)** | **Mozambique**  **N=280**  **n (%)** | **Sierra Leone**  **N=206**  **n (%)** | **South Africa**  **N=92**  **n (%)** |
| --- | --- | --- | --- | --- | --- | --- | --- | --- |
| Improved family planning | 305 (17) | 24 (7) | 247 (53) | 18 (7) | 2 (1) | 5 (2) | 7 (3) | 2 (2) |
| Improved antenatal care and obstetric care and management | 1532 (85) | 303 (83) | 392 (83) | 234 (94) | 108 (74) | 272 (97) | 149 (72) | 74 (80) |
| Improved infection prevention and control | 74 (4) | 5 (1) | 29 (6) | 15 (6) | 1 (1) | 21 (8) | 2 (1) | 1 (1) |
| Improved HIV prevention and control | 141 (8) | 6 (2) | 46 (10) | 60 (24) | 0 (0) | 3 (1) | 24 (12) | 2 (2) |
| Improved health-seeking behavior | 491 (27) | 56 (15) | 267 (57) | 93 (37) | 9 (6) | 13 (5) | 36 (17) | 17 (18) |
| Improved nutritional support | 100 (6) | 5 (1) | 92 (20) | 1 (0) | 0 (0) | 1 (0) | 1 (0) | 0 (0) |
| Improved vaccinations | 6 (0) | 1 (0) | 2 (0) | 0 (0) | 1 (1) | 2 (1) | 0 (0) | 0 (0) |
| Improved transport system | 66 (4) | 0 (0) | 42 (9) | 4 (2) | 4 (3) | 13 (5) | 2 (1) | 1 (1) |
| Other recommendation | 351 (19) | 66 (18) | 196 (42) | 55 (22) | 4 (3) | 5 (2) | 22 (11) | 3 (3) |
| *^1^Single case can have multiple recommendations. Includes deaths that are preventable given current situation or preventable given ideal situation.* | | | | | | | |  |
