# Supplementary material for: Causes of stillbirth in sub-Saharan Africa and South Asia: Findings from Child Health and Mortality Prevention Surveillance, 2016–2023
Source: PLOS Glob Public Health. 2026 Oct 27;6(10):e0005235. doi: 10.1371/journal.pgph.0005235 (PMC13712675; doi:10.1371/journal.pgph.0005235)
Supplement: Supplementary file 4 [file pgph.0005235.s002.docx]

**Reviewer’s Comment**

**General comments**

Thank you for inviting me to review this manuscript titled “Causes of Stillbirth in Sub-Saharan Africa and South Asia: Findings from the Child Health and Mortality Prevention Surveillance, 2016–2023.”

This paper addresses an important topic by examining the fetal and maternal causes of stillbirth in countries with a high disease burden. However, several issues require clarification and strengthening to improve the quality and interpretability of the manuscript.

**Country Selection and Representativeness**

Although it is clear that undertaking this kind of review is demanding, the diversity in healthcare quality, variability in disease prevalence across the two study regions, and differences in cultural practices suggest that the study outcomes could have been more generalizable if additional sites had been included. The review, however, did not sufficiently address this limitation.

For instance, based on 2015 estimates, Nigeria, one of the most populous nations in the world, contributed approximately 12.2% of global stillbirths. Furthermore, the 2024 UNICEF/UN Inter-Agency Report indicates that Nigeria recorded about 184,000 stillbirths in 2023, representing roughly 20% of all stillbirths in Sub-Saharan Africa (approximately 912,000). Given Nigeria’s substantial contribution to both the regional and global stillbirth burden, the complete absence of data from the country places a significant constraint on the generalisability of the findings.

It should therefore be explicitly stated that the results must be interpreted with caution, acknowledging this as a key limitation of the study.

Furthermore, it would be informative if the authors could clarify the proportion of stillbirth data obtained from community settings versus hospital-based facilities across the various study sites. This distinction is crucial for understanding potential selection bias and assessing the representativeness of the study population.

In addition, the authors are encouraged to present the key findings using infographics or other visual data summaries to enhance readability, facilitate comparative interpretation, and improve the overall clarity of data presentation.

**Introduction**

**Trend Projection and Target Achievement**

Could the authors project the estimated year when the global stillbirth reduction target might be achieved based on current trends? Including this analysis, even briefly in the abstract, would underscore the urgency for action and highlight the pace of progress.

**Methodological Details and results**

The Methods section is too brief and lacks sufficient detail for replication. Not all readers will have access to the referenced studies in the methods section. It would be helpful to provide a concise summary of the surveillance framework within the manuscript or include a graphical representation of the surveillance system to aid understanding. Describe the notification and reporting process used in the surveillance system.

Considering the wide variability in consent rates for MITS across study sites, there is a potential for differential data quality and possible misrepresentation of the true distribution of causes of death. To strengthen the validity of the findings, the authors are encouraged to apply appropriate statistical weighting or conduct sensitivity analyses when combining data from sites with markedly different consent proportions. This will help ensure more robust and generalisable conclusions.

The authors should recheck the results to ensure that the appropriate denominators were used in all calculations and data presentations.

**Clarifications**

What proportion of stillbirths were refrigerated prior to sampling?

What was the mean or median time from stillbirth to specimen collection at each site?

**Discussion**

The manuscript should briefly discuss any laboratory or logistical constraints, including instances of delayed sample collection, autolysis, or bacterial overgrowth that might affect the accuracy of MITS findings.

**References**

Self-Citation:

The manuscript contains too many self-citations. The authors should include additional relevant references from independent sources to ensure balance and enhance the credibility of the work.
