# Supplementary material for: Causes of stillbirth in sub-Saharan Africa and South Asia: Findings from Child Health and Mortality Prevention Surveillance, 2016–2023
Source: PLOS Glob Public Health. 2026 Oct 27;6(10):e0005235. doi: 10.1371/journal.pgph.0005235 (PMC13712675; doi:10.1371/journal.pgph.0005235)
Supplement: Peer review history

# Peer review: Decision Letter 0

**DOI:** 10.1371/journal.pgph.0005235.r001

**Authors:** Shiyam Sunder

26 Jan 2026

PGPH-D-25-02617

Causes of stillbirth in sub-Saharan Africa and South Asia: Findings from Child Health and Mortality Prevention Surveillance, 2016-2023

PLOS Global Public Health

Dear Dr. Gurley,

Thank you for submitting your manuscript to PLOS Global Public Health. After careful consideration, we feel that it has merit but does not fully meet PLOS Global Public Health’s publication criteria as it currently stands. Therefore, we invite you to submit a revised version of the manuscript that addresses the points raised during the review process.

Please submit your revised manuscript by Mar 12 2026 11:59PM. If you will need more time than this to complete your revisions, please reply to this message or contact the journal office at globalpubhealth@plos.org. When you're ready to submit your revision, log on to https://www.editorialmanager.com/pgph/ and select the 'Submissions Needing Revision' folder to locate your manuscript file.

Please include the following items when submitting your revised manuscript:

- A letter that responds to each point raised by the editor and reviewer(s). You should upload this letter as a separate file labeled 'Response to Reviewers'.
- A marked-up copy of your manuscript that highlights changes made to the original version. You should upload this as a separate file labeled 'Revised Manuscript with Track Changes'.
- An unmarked version of your revised paper without tracked changes. You should upload this as a separate file labeled 'Manuscript'.

Guidelines for resubmitting your figure files are available below the reviewer comments at the end of this letter.

We look forward to receiving your revised manuscript.

Kind regards,

Shiyam Sunder, MBBS, MSc epidemiology, PhD

Academic Editor

PLOS Global Public Health

Journal Requirements:

If the reviewer comments include a recommendation to cite specific previously published works, please review and evaluate these publications to determine whether they are relevant and should be cited. There is no requirement to cite these works unless the editor has indicated otherwise.

Additional Editor Comments (if provided):

[Note: HTML markup is below. Please do not edit.]

Reviewers' comments:

Reviewer's Responses to Questions

**Comments to the Author**

1. Does this manuscript meet PLOS Global Public Health’s publication criteria?

Reviewer #1: Yes

Reviewer #2: Yes

**********

2. Has the statistical analysis been performed appropriately and rigorously?

Reviewer #1: Yes

Reviewer #2: No

**********

3. Have the authors made all data underlying the findings in their manuscript fully available (please refer to the Data Availability Statement at the start of the manuscript PDF file)?

The PLOS Data policy

Reviewer #1: Yes

Reviewer #2: Yes

**********

4. Is the manuscript presented in an intelligible fashion and written in standard English?

Reviewer #1: Yes

Reviewer #2: Yes

**********

Reviewer #1: Reviewer’s Comment

General comments

Thank you for inviting me to review this manuscript titled “Causes of Stillbirth in Sub-Saharan Africa and South Asia: Findings from the Child Health and Mortality Prevention Surveillance, 2016–2023.”

This paper addresses an important topic by examining the fetal and maternal causes of stillbirth in countries with a high disease burden. However, several issues require clarification and strengthening to improve the quality and interpretability of the manuscript.

Country Selection and Representativeness

Although it is clear that undertaking this kind of review is demanding, the diversity in healthcare quality, variability in disease prevalence across the two study regions, and differences in cultural practices suggest that the study outcomes could have been more generalizable if additional sites had been included. The review, however, did not sufficiently address this limitation.

For instance, based on 2015 estimates, Nigeria, one of the most populous nations in the world, contributed approximately 12.2% of global stillbirths. Furthermore, the 2024 UNICEF/UN Inter-Agency Report indicates that Nigeria recorded about 184,000 stillbirths in 2023, representing roughly 20% of all stillbirths in Sub-Saharan Africa (approximately 912,000). Given Nigeria’s substantial contribution to both the regional and global stillbirth burden, the complete absence of data from the country places a significant constraint on the generalisability of the findings.

It should therefore be explicitly stated that the results must be interpreted with caution, acknowledging this as a key limitation of the study.

Furthermore, it would be informative if the authors could clarify the proportion of stillbirth data obtained from community settings versus hospital-based facilities across the various study sites. This distinction is crucial for understanding potential selection bias and assessing the representativeness of the study population.

In addition, the authors are encouraged to present the key findings using infographics or other visual data summaries to enhance readability, facilitate comparative interpretation, and improve the overall clarity of data presentation.

Introduction

Trend Projection and Target Achievement

Could the authors project the estimated year when the global stillbirth reduction target might be achieved based on current trends? Including this analysis, even briefly in the abstract, would underscore the urgency for action and highlight the pace of progress.

Methodological Details and results

The Methods section is too brief and lacks sufficient detail for replication. Not all readers will have access to the referenced studies in the methods section. It would be helpful to provide a concise summary of the surveillance framework within the manuscript or include a graphical representation of the surveillance system to aid understanding. Describe the notification and reporting process used in the surveillance system.

Considering the wide variability in consent rates for MITS across study sites, there is a potential for differential data quality and possible misrepresentation of the true distribution of causes of death. To strengthen the validity of the findings, the authors are encouraged to apply appropriate statistical weighting or conduct sensitivity analyses when combining data from sites with markedly different consent proportions. This will help ensure more robust and generalisable conclusions.

The authors should recheck the results to ensure that the appropriate denominators were used in all calculations and data presentations.

Clarifications

What proportion of stillbirths were refrigerated prior to sampling?

What was the mean or median time from stillbirth to specimen collection at each site?

Discussion

The manuscript should briefly discuss any laboratory or logistical constraints, including instances of delayed sample collection, autolysis, or bacterial overgrowth that might affect the accuracy of MITS findings.

References

Self-Citation:

The manuscript contains too many self-citations. The authors should include additional relevant references from independent sources to ensure balance and enhance the credibility of the work.

Reviewer #2: Overall assessment

This is a large, multi-country analysis of CHAMPS stillbirth investigations using MITS and a standardized DeCoDe process across seven sites (2016–2023), with high cause-of-death ascertainment (94%) and an explicit “missed prevention opportunities” component. The dataset scale and standardized postmortem approach are major strengths and highly relevant for stillbirth prevention in high-burden settings.

Major comments

The paper reports that 56% of eligible families consented to MITS, with wide site variation (e.g., 32% Bangladesh vs 82% Kenya). This raises concerns about selection bias and comparability across sites. Provide a comparison of MITS-consented vs non-consented eligible stillbirths (where available): maternal age, location (facility/community), maceration status, gestational age/weight, timing since death notification, etc. and explicitly discuss how consent variation could influence the observed distribution of causes (e.g., facility vs community deaths, differences in availability of clinical records/placenta).

“Intrauterine hypoxia” is assigned as the main fetal condition in 75% overall, and remains dominant across sites. Because this category can function as a “final common pathway,” readers will ask how often it is a specific diagnosis versus a label used when no fetal pathology is identified. Provide a clear operational definition in the main text (not only referenced standards): how panels distinguish hypoxia due to intrapartum events, maternal hypertension, placental abruption, cord events, etc. and Quantify how often hypoxia is assigned with identified upstream maternal/placental conditions vs unexplained (you mention 18% without underlying maternal/placental causes bring this into the main Results narrative with a short interpretation). Consider presenting hypoxia stratified by timing (antepartum vs intrapartum) where timing is known, since prevention differs substantially.

There is notable missingness: gestational age unknown for 21%, timing unknown for 24%, placenta unavailable for 51%, and fetal heartbeat documentation missing in >50% of cases. Add a dedicated missingness table (overall and by site) for the key variables that drive classification and prevention recommendations (GA, timing, fetal heart monitoring, placenta availability, completeness of maternal records). Explicitly describe whether and how missingness influenced DeCoDe confidence levels (you note level-1 confidence for fetal condition in 81%; show how this varies by site and by major cause group). Consider sensitivity analyses restricted to: 1) cases with placenta examined; and/or, 2) cases with known timing; and/or, 3) cases with GA known (for SGA-related analyses).

You report 72% of stillbirths are considered preventable, and that 85% of preventable deaths could be prevented via improved antenatal/obstetric care. This is policy-relevant but vulnerable to subjectivity and site-to-site interpretation differences. Define “preventable” explicitly: preventable under “optimal provision of existing care”—what constitutes “existing” across sites with different resources? Provide information on standardization and inter-panel consistency: 1) training, decision rules, and whether cross-site calibration occurred; and 2) whether a subset of cases was double-reviewed across sites or centrally adjudicated. Present preventability by cause group (hypoxia, congenital infection, congenital anomalies, etc.) and by timing (antepartum/intrapartum), because prevention levers differ. The subcategory expansion (more granular prevention categories) is good please add the actual category definitions in a supplement and summarize the top 5 subcategories in the main text for readability.

In Methods, there is a sentence about exploring the relationship between foot length and gestational age and distributions for SGA estimation. This reads like an editing artifact and is confusing in a stillbirth MITS/DeCoDe paper. Remove this sentence unless it is central to your GA/SGA classification approach. If you keep it, clarify: Why was foot length used here? Was GA sometimes derived from anthropometry? If yes, specify the algorithm and validation, and how it impacts SGA classification.

You identify multiple pathogens using TAC PCR and cultures, and list major pathogens (GBS, E. coli, Enterococcus). Since MITS-based pathogen detection can be sensitive, readers will want reassurance around contamination, colonization vs invasive infection, and causal inference. State the causal criteria for assigning congenital infection as cause (e.g., pathogen in sterile site + histopathology consistent with infection + clinical context). Describe contamination control steps for sampling and lab workflows, and how you handled polymicrobial detections (not uncommon in your data). Consider providing a breakdown of congenital infections by sterile-site vs non-sterile-site detection and by confidence level.

Minor comments

The Findings are clear and compelling; consider adding a one-line note on consent rate and missingness to prevent overinterpretation of representativeness.

Stillbirth definition is provided (≥1000g or ≥28 weeks) and death notification window described; please also specify whether multiple births were included and how they were handled.

Statistical analysis is described briefly; add which comparisons were planned a priori, and consider adjusting for multiple comparisons if many site-wise tests are presented.

Ethics statement is concise; consider adding a sentence acknowledging cultural sensitivity around MITS and how community engagement supported consent processes (you cite that methods exist elsewhere).

Table 1 is heavily relied upon; ensure it includes denominators for each key variable (e.g., fetal heartbeat documentation) and site-stratified missingness.

For the statement that 95% delivered in a healthcare facility, discuss how CHAMPS case ascertainment might skew toward facility stillbirths and how community surveillance complements this.

You mention 32 maternal deaths in clinical records without cause information—either remove or briefly interpret (e.g., indicates gaps in maternal chart documentation) and consider whether maternal death should be integrated into the causal chain where possible.

The discussion appropriately emphasizes geographic heterogeneity and care gaps.

Consider strengthening the “what next” section with 2–3 priority, context-specific interventions mapped to the largest preventable fractions by region/site-type (even if kept high level).

When stating that congenital infections may be higher in South Africa because care quality is better (thus fewer deaths from care gaps), frame this explicitly as a hypothesis and note alternative explanations (e.g., differential specimen handling, diagnostic intensity, panel tendencies).

The CHAMPS data access statement is included; consider stating what elements are publicly accessible vs require request, and whether de-identified MITS/pathology results are shareable.

**********

what does this mean?). If published, this will include your full peer review and any attached files.

**Do you want your identity to be public for this peer review?**  If you choose “no”, your identity will remain anonymous but your review may still be made public.

For information about this choice, including consent withdrawal, please see our Privacy Policy

Reviewer #1: **Yes:**  Alao Michael Abel

Reviewer #2: No

**********

[NOTE: If reviewer comments were submitted as an attachment file, they will be attached to this email and accessible via the submission site. Please log into your account, locate the manuscript record, and check for the action link "View Attachments". If this link does not appear, there are no attachment files.]

Attachment Submitted filename: Reviewer.docx

# Author comment: Author response to Decision Letter 1

**DOI:** 10.1371/journal.pgph.0005235.r002

17 May 2026

Attachment Submitted filename: Response to Editorial Comments_17 May 2026_AR_Clean.docx

# Peer review: Decision Letter 1

**DOI:** 10.1371/journal.pgph.0005235.r003

**Authors:** Nicola L. Hawley

4 Aug 2026

Causes of stillbirth in sub-Saharan Africa and South Asia: Findings from Child Health and Mortality Prevention Surveillance, 2016-2023

PGPH-D-25-02617R1

Dear Dr. Gurley,

We are pleased to inform you that your manuscript 'Causes of stillbirth in sub-Saharan Africa and South Asia: Findings from Child Health and Mortality Prevention Surveillance, 2016-2023' has been provisionally accepted for publication in PLOS Global Public Health.

Before your manuscript can be formally accepted you will need to complete some formatting changes, which you will receive in a follow up email. A member of our team will be in touch with a set of requests.

Please note that your manuscript will not be scheduled for publication until you have made the required changes, so a swift response is appreciated.

IMPORTANT: The editorial review process is now complete. PLOS will only permit corrections to spelling, formatting or significant scientific errors from this point onwards. Requests for major changes, or any which affect the scientific understanding of your work, will cause delays to the publication date of your manuscript.

If your institution or institutions have a press office, please notify them about your upcoming paper to help maximize its impact. If they'll be preparing press materials, please inform our press team as soon as possible -- no later than 48 hours after receiving the formal acceptance. Your manuscript will remain under strict press embargo until 2 pm Eastern Time on the date of publication. For more information, please contact globalpubhealth@plos.org.

Thank you again for supporting Open Access publishing; we are looking forward to publishing your work in PLOS Global Public Health.

Best regards,

Nicola L. Hawley

Academic Editor

PLOS Global Public Health

***********************************************************

Reviewer Comments (if any, and for reference):

Reviewer's Responses to Questions

**Comments to the Author**

Reviewer #1: All comments have been addressed

**********

publication criteria?

Reviewer #1: Yes

**********

3. Has the statistical analysis been performed appropriately and rigorously?

Reviewer #1: Yes

**********

4. Have the authors made all data underlying the findings in their manuscript fully available (please refer to the Data Availability Statement at the start of the manuscript PDF file)?

The PLOS Data policy

Reviewer #1: Yes

**********

5. Is the manuscript presented in an intelligible fashion and written in standard English?

Reviewer #1: Yes

**********

Reviewer #1: The authors have carefully addressed my comment to my satisfaction. I have no further comments.

**********

what does this mean?). If published, this will include your full peer review and any attached files.

**Do you want your identity to be public for this peer review?**  If you choose “no”, your identity will remain anonymous but your review may still be made public.

For information about this choice, including consent withdrawal, please see our Privacy Policy

Reviewer #1: **Yes:**  Alao Micheal Abel

**********
